# A comprehensive study of recent maximum power point tracking techniques for photovoltaic systems

**DOI:** 10.1038/s41598-025-96247-5

**Published:** 2025-04-24

**Authors:** Mohammed Hamouda Ali, Mohammad Zakaria, Sally El-Tawab

**Affiliations:** Department of Electrical Engineering, Faculty of Engineering, Al-Azhar University, Cairo, 11651 Egypt

**Keywords:** PV system, MPPT, Perturb and observation (P&O), Particle swarm optimization (PSO), Incremental conductance (INC), Artificial intelligence (AI), MCDM, Engineering, Electrical and electronic engineering

## Abstract

The percentage of renewable energy in the global mix of energy sources is rising annually, with solar photovoltaics (PVs) accounting for most capacity expansions due to their widespread availability, safety, and cleanliness. Because the amount of energy generated is limited by the poor efficiency of the photovoltaic cells and the characteristics of the connected load and weather fluctuation, maximum power point tracking (MPPT) strategies are crucial for maximizing the power delivered in PV production systems. These MPPT techniques face several issues and limitations, so this paper has focused more on modeling and developing the MPPT techniques in PV systems. The MPPT-based methodologies fall into three categories: artificial intelligence (AI), metaheuristic, and conventional. Five of these techniques have been proposed here to solve the MPPT problem. The perturb & observe (P&O) and incremental conductance (INC) methods have been used as conventional methods. In contrast, particle swarm optimization (PSO) has been used as a metaheuristic method. Finally, the artificial neural network (ANN) and fuzzy logic control (FLC) techniques have been used as AI methods. Each technique is analyzed critically in terms of tracking speed, algorithm complexity, and dynamic tracking in different environmental conditions. Furthermore, this comprehensive study of MPPT methods aims to be a guideline for selecting the best MPPT method for optimal operation under the environmental conditions of PV systems by employing multi-criteria decision-making (MCDM) based on AHP and CRITIC weighting methods, as well as the ranking method (VIKOR), to compare and rank the MPPT methods based on their effectiveness and economic feasibility. The results show AI techniques have a tracking efficiency of almost 99% when compared to other examined approaches, and they give quick and efficient tracking speed.

## Introduction

The energy produced from fossil fuels represents more than 80%^1^, which has serious impacts on the environment, such as global warming, drought, floods, storms, and rising sea levels^2^. Overcoming these problems has become the focus of great attention for the global market. Therefore, renewable energy has been mainly employed to produce electricity all over the world^3^. One of the greatest renewable energy sources is solar energy, which comes from the sun directly and is pure energy that doesn’t hurt the environment. Every day, the sun produces energy that is around a thousand times more than what is generated from fossil fuels^4^. Solar energy may be utilized to generate power in practically all countries without relying on other countries because it is an endless resource that guarantees its long-term usage. PV systems are widely available, long-lasting, silent, and clean. They also have no rotating elements and are employed in a variety of applications, including solar-powered vehicles, hybrid energy systems, and roadway illumination^5^. By 2050, photovoltaic systems are expected to provide around 11% of global power and prevent 2.3 gigatons of CO2 emissions annually, according to the International Energy Agency^6^.

Four key parts make up PV systems, which are straightforward to utilize in residential and commercial settings: batteries, charge controllers, DC/AC converters, and PV panels. The quantity of energy generated by solar panels can be lowered by several fundamental variables, including temperature variation, solar irradianceintensity, dust, geographical location, and cloud cover. Every PV panel has a different output of electricity based on its resistive load, which reduces power production even at the same temperatures and irradiance^7^. The two types of solar charge controllers that are most used are those that employ maximum power point tracking (MPPT) and pulse width modulation (PWM). Within the off-grid solar businesses, both methods are frequently employed. To provide a consistent output voltage, the PWM controls modify the duty ratio of the switches in response to changes in the input. A square-wave signal that alternates between completely on and zero is created from the DC voltage. Digital encoding of analog signal levels is accomplished by PWM. In addition, it offers trickle charging and regulates the current used to charge the battery. The primary function of the MPPT charge controller is to get the most power output from the PV module^8^. The principal objective of the MPPT mechanism is to sample the PV array’s output and implement the proper resistance to get the greatest power under any specific combination of environmental circumstances. MPPT-equipped controllers offer several benefits over PWM controllers, including the following^9^:

1. Better efficiency.
2. The ability to optimize DC load in addition to voltage differences.
3. Suitable for more complex systems where significantly higher solar panel output than battery voltage.
4. Improves the system’s production, which raises its capacity.

Addressing the charge controller in photovoltaic (PV) systems is crucial, as it is a key component in ensuring the efficient, safe, and reliable operation of solar energy setups. The benefits of using a proper charge controller can be listed as (a) boosting system efficiency; (b) battery longevity; (c) improved reliability; (d) safety assurance; and (e) scalability. However, occasionally, it might be challenging to determine the precise maximum power point (MPP) because of the PV sources’ non-linear current-voltage (I-V) characteristics and the result of the fluctuating ambient circumstances. Numerous MPPT methods, like conventional algorithms, artificial intelligence (AI), and meta-heuristics, can be used to address this problem^10^. There are differences between these algorithms in terms of reaction time, tracking speed, and complexity. Conventional algorithms are the most widely used because of their ease of use, quickness of response, and simplicity of implementation. Perturb & Observe (P&O), Incremental Conductance (INC), Incremental Resistance (INR), Fractional Open-Circuit Voltage (FOCV), and Fractional Short-Circuit Current (FSCC) are the most often used traditional algorithms. PV-MPPT frequently uses the P&O approach, yet it has several drawbacks, including drift concerns linked to rapidly changing irradiance, high oscillation, and poor tracking speed^11^. As a result, the INC-MPPT was developed to address the P&O method’s inadequacies. The main advantage of this method is its capacity to achieve the MPP in quickly altering environmental conditions^12^. However, because a PV system operates via a derivative operation, imbalance and measurement mistakes provide a significant challenge. As a result, several adjustments have been made to overcome the difficulties with ICN-MPPTs and P&O^11–14^, but they are still considered inadequate solutions.

On the other hand, approaches based on AI have been put out to circumvent these problems because they don’t call for exact parameters or complex mathematical computations to be made to build systems. The artificial neural network (ANN)^15^ and the FLC^16^ are the main components of these methods. Moreover, they offer more accurate and adaptable control, especially for non-linear systems. The FLC’s superior tracking speed and reduced oscillation when compared to traditional MPPT techniques give it a highly efficient rating as a PV system controller^17^. However, the problem of choosing suitable membership functions remains unresolved. The ANN is regarded as another efficient method for non-linear systems such as PV modules. It uses the quantification of the actual numerical data to provide heuristic output. However, the main shortcomings of the ANN system are its slow training and black-box operation^18^. When it comes to combining the learning powers of ANN with the ability of FLC to deal with erroneous input, the ANN plus FLC combination appears more appealing and appropriate for PV applications^19^. However, acquiring a large amount of training data is the main challenge in constructing the adaptive neuro-fuzzy inference system (ANFIS) MPPT controller. In^20^, ANFIS-MPPT training was carried out on experimental data. However, the experimental data has several serious flaws, including a small dynamic range. Moreover, errors are common in practical data collection, and the data collected is limited to a specific geographic area. The PV module was stimulated to provide the training dataset. The suggested method was more complex since it suggested two steps to determine the duty cycle.

The ability of MPPT based on metaheuristic approaches to address the nonlinear I-V or power voltage (P-V) functions problem has garnered significant attention from research groups in recent years^10^. Among these approaches, the most recent ones that have been suggested are listed as follows: ABC, SSA, JA, FSSO, GWO, EPO, CSA, FA, in addition to ACO and PSO. Our attention was drawn to the PSO algorithm’s utilization in the PV system industry due to its potential to achieve optimal performance and flexibility in a variety of applications. Table 1 provides an overview of the prior methods used to solve the MPPT problem. The categories, methods, contributions, main findings, and weaknesses of each application can be used to categorize these approaches.

**Table 1:** Summary of the proposed MPPT methods in literature.

Class.	MPPT tech.	Conv.	Contribution	Demerits	Ref. no	Year
Conventional	INC	Boost	This study modified the incremental conductance algorithm to increase the speed of MPP tracking.	The primary drawback of this study is that it considers the change in solar radiation while cell temperature is constant.	^21^	2024
	P&O	Buck-Boost	P&O performance for resistive and motor-pump loads at high perturbation rates, uniform, and quickly changing irradiance was verified.	Even when both irradiance and cell temperature are constant, system waveforms oscillate about their MPP values because of the P&O algorithm’s ongoing disturbances.	^22^	2015
	Hill climbing (HC)	Buck-Boost	These methods, which have been tried and proven in actual weather scenarios, employ the boost converter’s duty cycle as a feedback parameter when the MPPT task is being performed.	The primary drawback of this method stems from the trade-off between the system’s stability throughout a continuous irradiance period and its inability to react quickly in the event of a sudden shift in irradiance.	^23^	2019
Meta-heuristic	MPSO	Boost	A modified PSO technique is used to minimize computational time, accomplish cost optimization, and improve the performance of the MPP for the PV array.	Minor adjustments in the duty cycle may cause the MPP search to run more slowly. Major fluctuations may occur while supplying an optimal search solution, which results in large computational costs and energy waste.	^24^	2020
	OBEO	Boost	To track the GMPP in the PV system under varied partial shading situations, the OBEO algorithm is capable of balancing exploration and exploitation in a shorter convergence time.	Because the test circumstances are like the temperatures in tropical nations, they are relevant to equatorial countries.	^25^	2022
	ICSA	Buck-Boost	With the least number of steady-state oscillations, the smallest convergence time, and the minimum failure rate, this technique enhances the tracking mechanism of PV systems to track their GMPP for uniform irradiance and PSC.	High oscillations surrounding the conditions of steady state.	^26^	2021
	IGWO	Buck-Boost	GWO exhibits faster convergence compared to traditional techniques like Perturb and Observe (P&O), resulting in quicker achievement of the MPP and reduced energy loss.	GWO requires more computation compared to simpler MPPT algorithms; the performance of GWO can be sensitive to the selection of its control parameters.	^27^	2021
Artificial intelligent	FLC	Boost	Fuzzy logic controllers can react quickly to changes in operating conditions, ensuring the system stays close to the MPP (Maximum Power Point) even during rapid fluctuations.	Fuzzy logic requires defining membership functions and fuzzy rules, which can be complex and time-consuming to design and optimize.	^16^	2018
	ANFIS	Boost	A fitting curve technique was used to examine and optimize the enormous training dataset. The maximum PV power was the ANFIS model’s output, and the solar irradiance and ambient temperature were chosen as the inputs. Two steps have been used to determine the duty cycle: First, under the identical climatic conditions, the output of the ANFIS model was compared with the actual PV power, which was measured using a PV Simulink model. The fault was then passed to a PI controller, which used the PWM generator to create a signal for a DC–DC converter to modify the PV array’s operational MPP.	Large training datasets demand considerable processing time, a lot of memory, and previous training. Furthermore, the data collected is restricted to a particular geographic area, and errors are frequent in practical data collecting. To supply the training dataset, the PV module was prompted. Because it proposed two stages to establish the duty cycle, the suggested approach was more complicated.	^20^	2019

In this paper, different approaches have been introduced to address the challenges in solving the MPPTproblem. This paperpresents several methods to overcome the demerits of conventional methods in the form of metaheuristic and AI methods. We apply all ways to solve conventional MPPT issues on the same PV module under the same environmental circumstances to validate the efficacy of our suggested approaches. Our contributions include modeling and testing the characteristics of PV modules and providing several methods, comparing, and demonstrating their effectiveness in the MPPT problem. The amalgamation of these contributions offers a complete and inventive resolution to augment power system optimization. The following items encapsulate the primary contributions of this work:

1. Provide an extensive review of traditional MPPT strategies and try to review other global MPPT strategies.
2. Verify and validate the PV modeling and analyze the PV performance under different environmental conditions before applying the MPPT methodology.
3. Propose the PWM and PWM-PI charge controllers to discover which has the best cost-benefit ratio in similar environments.
4. Applying five recent and effective algorithms and finding one suitable algorithm for all study cases: Perturb and Observe (P&O), Particle Swarm Optimization (PSO), Incremental Conductance (IC), Artificial Neural Networks (ANN), and Fuzzy Logic Control (FLC).
5. The performances of proposed methods have been compared based on desirable features such as time complexity, range of effectiveness, traceability under changing environmental conditions, stability, power ripple, and under- and overshooting of power.
6. A subjective and objective MCDM has been utilized to select the most suitable method for solving the MPPT problem and enhancing the performance and effectiveness of the PV system.
7. This is the first time a hybrid AHP-CRITIC approach has been introduced to determine the weights of different criteria for using it for ranking MPPT methods.

This paper is organized as follows: The second section offers a succinct outline of photovoltaic system modeling. Section 3 describes the different methods to design the MPPT controller. Section 4 introduces the MPPT mathematical formulation model. The objective and subjective weights of various criteria for using it for ranking MPPT methods are presented in Sect. 5. Section 6 contains a presentation and discussion of the simulation results. Lastly, part 7 brings the research to a close and mentions the gaps and recommendations for future work.

## PV systemmodel and characteristics

**Fig. 1:**
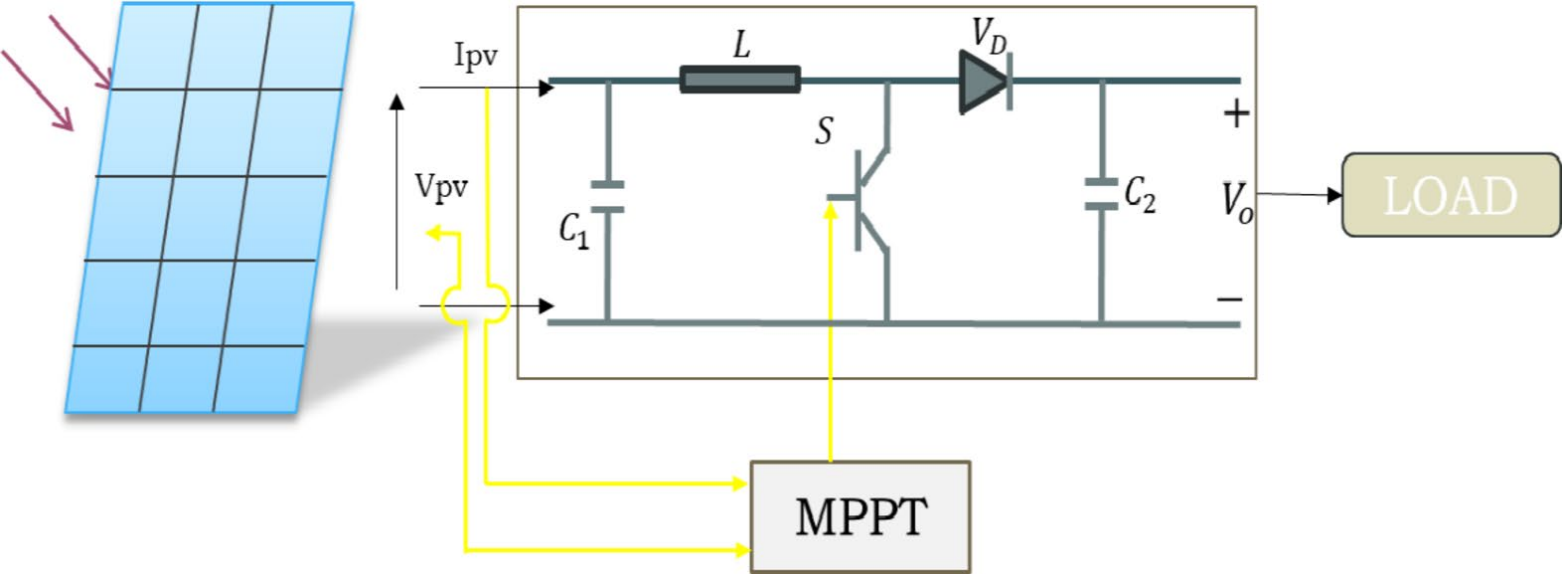
PV system with MPPT controller and boost converter.

Figure 1 shows the basic layout of the suggested PV system. It is formed up of an MPPT controller, a load, a boost converter, and a PV module for extracting solar energy. The PV arrays are constructed using parallel strings, each of which is made up of a few modules connected in series. Typically, a single module is constructed using cells connected in series. Because a single PV cell can only provide a small amount of power, many cells must be assembled to feed different PV applications. PV cells use the optoelectronic phenomenon known as the photovoltaic effect to generate electricity from light energy. Crystalline silicon, which can be taken out of rock or sand, is the material most used in solar cells. PV modules are essentially manufactured using monocrystalline, polycrystalline, or amorphous technologies.

### PV cell modeling

**Fig. 2:**
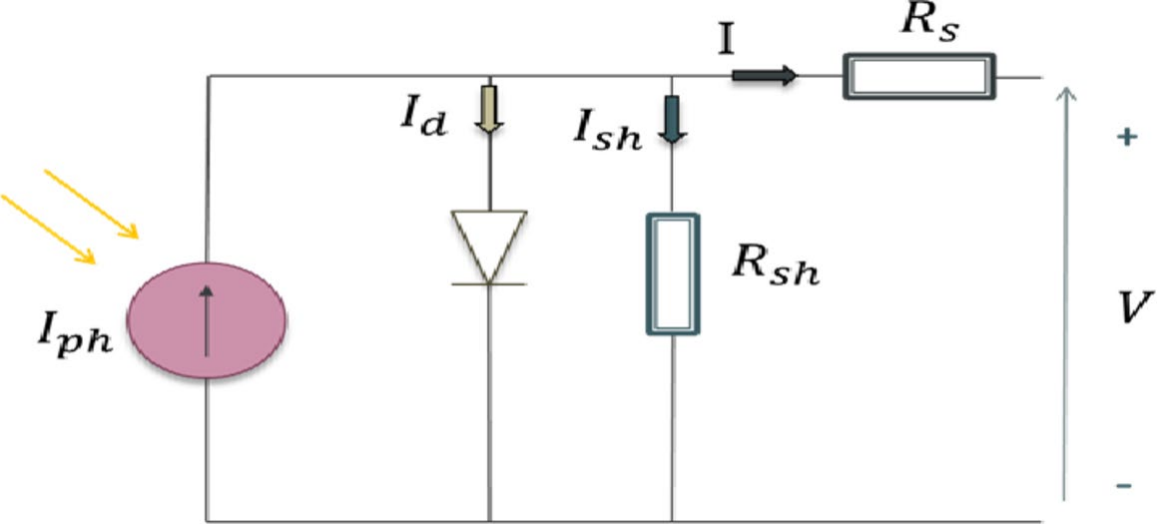
Equivalent circuit of PV module.

Exponential models, either single or double, are commonly used to depict PV cells; however, the double model is more challenging to solve. In this research, an effective compromise is made between accuracy and model complexity by utilizing the paradigm of a single exponential in Fig. 2 for simplicity^16^. A diode is linked in an anti-parallel fashion to the light-generated current source.The circuit of the single diode model has five parameters: photovoltaic current ($\:{I}_{ph}$), ideality factor ($\:n$) of the diode, saturation current of the diode ($\:{I}_{0}$), series resistance ($\:{R}_{s}$)which expresses the drop in the voltage, and shunt resistance ($\:{R}_{sh}$)which expresses the drop in the current.

**Fig. 3:**
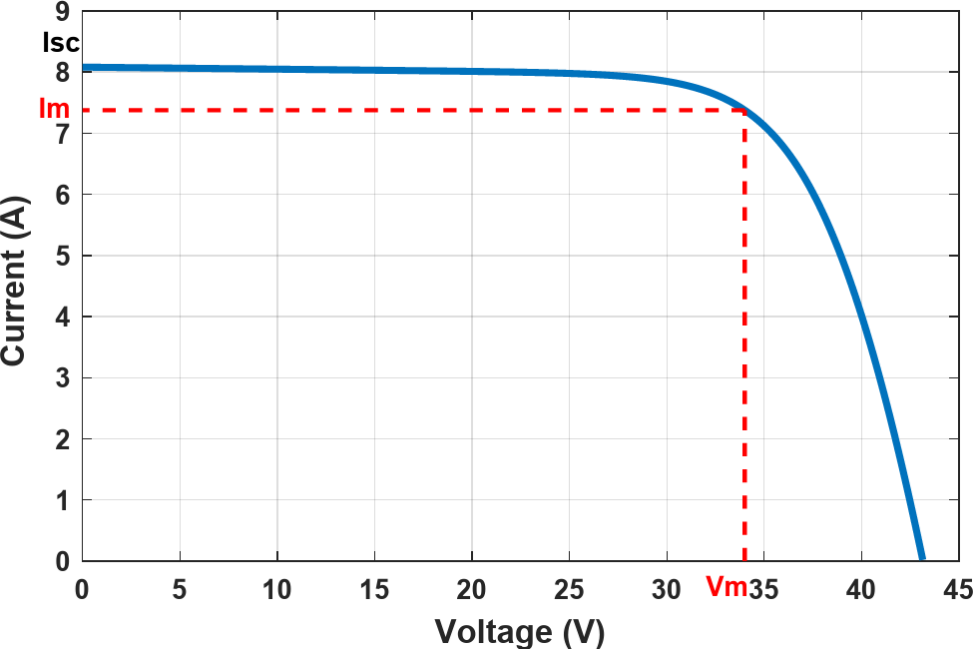
The I-V characteristic at STC.

Sunlight is constructed by photons. When those photons fall into semiconductor cells, the electrons will absorb the energy in those photons and be set free to move and produce DC flow in the external circuit according to Eq. (1).

1 $$\:I={I}_{ph}-{I}_{o}\left({e}^{\left(\frac{q\left(V+I*{R}_{s}\right)}{K*T*n*{N}_{s}}\right)}-1\right)-\frac{\left(V+I*{R}_{s}\right)}{{R}_{sh}}$$

2 $$\:{I}_{o}={I}_{rs}{\left(\frac{T}{{T}_{n}}\right)}^{3}*{e}^{\left(\frac{q*{E}_{go}}{nk}\right)\left(\frac{1}{{T}_{n}}\:-\:\frac{1}{T}\right)}$$

3 $$\:{I}_{rs}=\frac{{I}_{sc}}{{e}^{\left(\frac{q*{V}_{oc}}{n*{N}_{s}*K*T}\right)}-1}$$

where $\:T$stands for Kelvin’s temperature,$\:\:q$ for the electron’s charge, $\:K$ for the Boltzmannconstant,$\:{E}_{go}$represents the band gap for silicon$\:,{N}_{s}$number of series-connected cells, and the PV panel’s current and voltage are represented by $\:I$ and $\:V$, respectively.It is important to remember that the produced photovoltaic current, or $\:{I}_{ph}$, is determined by the surrounding circumstances and is expressed by the following expression^28^.

4 $$\:{I}_{ph}=({I}_{sc}+{K}_{i}*\left(T-{T}_{n}\right))*\frac{G}{1000}$$

where $\:{T}_{n}$stands for the values of temperature and$\:{I}_{sc}$ for the PV current under thestandard test conditions (STC). There is 1000 W/m^2^ of irradiance and a temperature of 25 °C at STC^18^. Symbol $\:{K}_{i}$represents the temperature coefficient of photocurrent.Table 2 displays the detailed electrical characteristics of the PV panel used in this investigation at STC. Seldom do actual outdoor circumstances meet STC requirements. Consequently, the expansion of solar irradiance is related to the PV system’s power supply, resulting in a negative linear relationship between output power and temperature fluctuations. PV cells have many appealing qualities, but they continue to have poor energy efficiency. The PV cell’s nonlinear I-V and P-V characteristics are highly dependent on the previously discussed variables about the surrounding environment, such as irradiance and temperature. The complicated link between temperature and total resistance in solar cells is illustrated in Fig. 3, which displays the I-V characteristics of non-linear output efficiency.

**Table 2:** PV panel specifications at STC.

No.	Parameters	Value
1	$\:{V}_{m}$	34.04 V
2	$\:{I}_{m}$	7.4171 A
3	$\:{P}_{m}$	252.4781 W
4	$\:{V}_{oc}$	43.2 V
5	$\:{I}_{sc}$	8.09 A
6	$\:{R}_{s}$	0.3353 Ω
7	$\:{R}_{sh}$	297.9752 Ω
8	$\:n$	1.3898
9	$\:{K}_{i}$	0.00102 A/C°
10	$\:{T}_{n}$	298 C°
11	$\:{E}_{go}$	1.1 eV
12	$\:{N}_{s}$	72

**Fig. 4:**
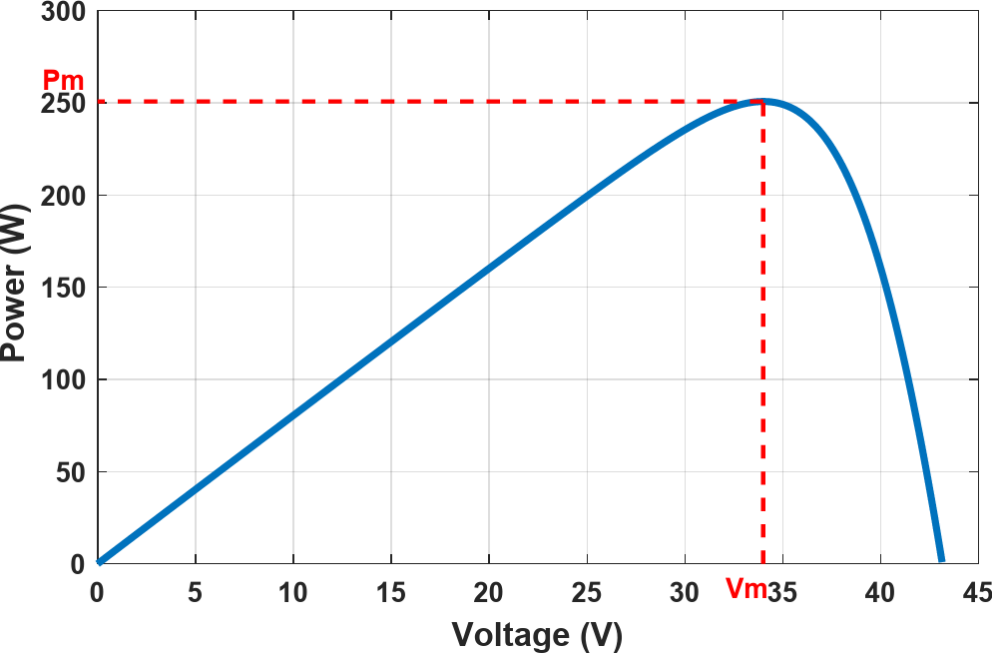
The P-V characteristic at STC.

The effects of uniform solar irradianceare seen in Figs. 3 and 4, where the PV cellhas a single operating point known as the MPP, where the maximum voltage$\:{V}_{m}$ meets the maximum current$\:{I}_{m}$. Because of this, it might be challenging to extract the maximum power output of the solar cell in unpredictable atmospheric circumstances. To maximize efficiency as well as generate and transmit as much power as possible from the solar cell to the load, a sophisticated control strategy known as MPPT is required. Furthermore, the functioning of the PV system employing the MPPT approach results in a high power output. As a result, it reduces the total quantity of PV cells required, which lowers the overall cost.

**Fig. 5:**
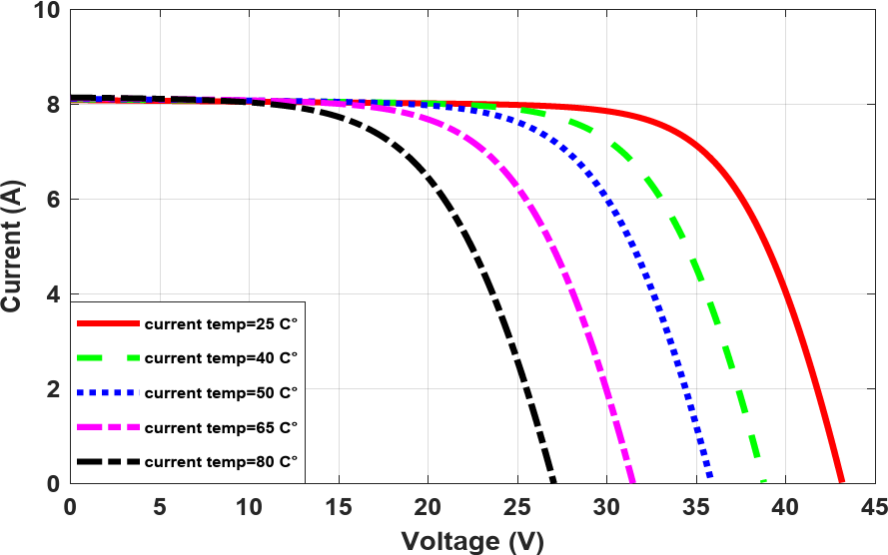
The I-V characteristic at different temperatures.

**Fig. 6:**
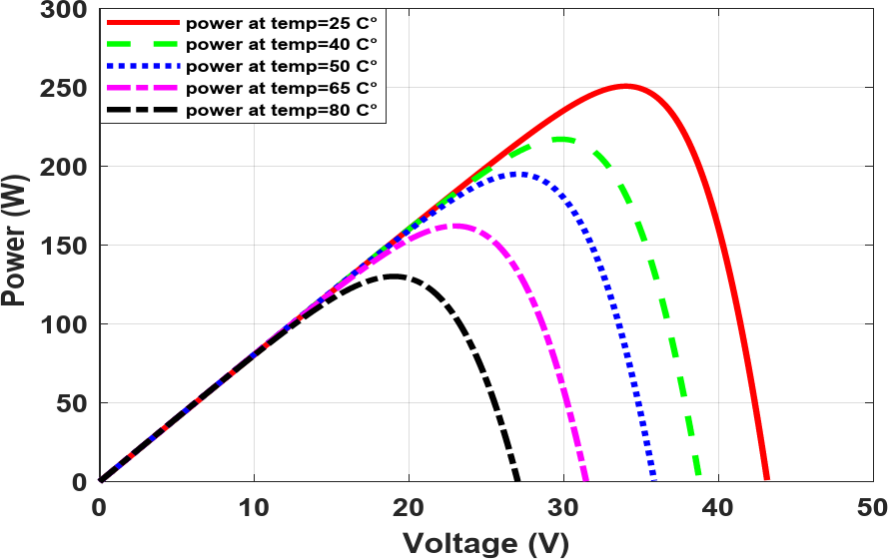
The P-V characteristic at different temperatures.

**Fig. 7:**
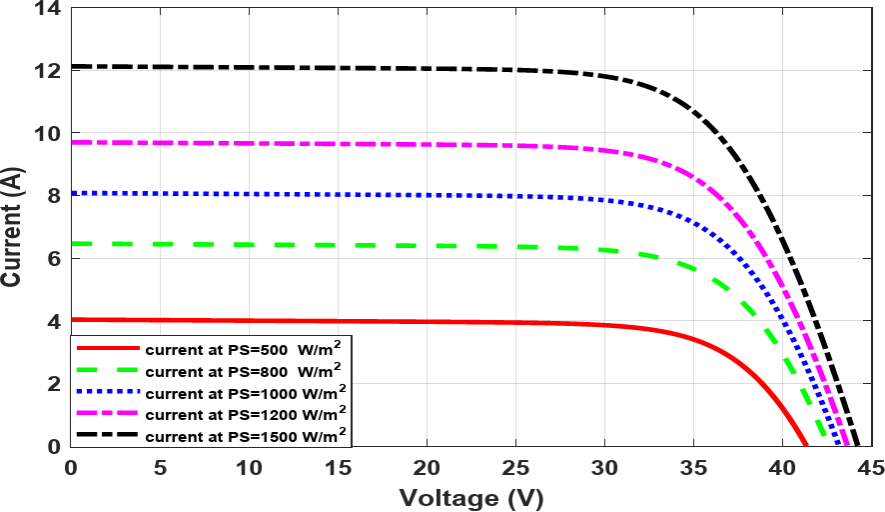
The I-V characteristic at different irradiance.

**Fig. 8:**
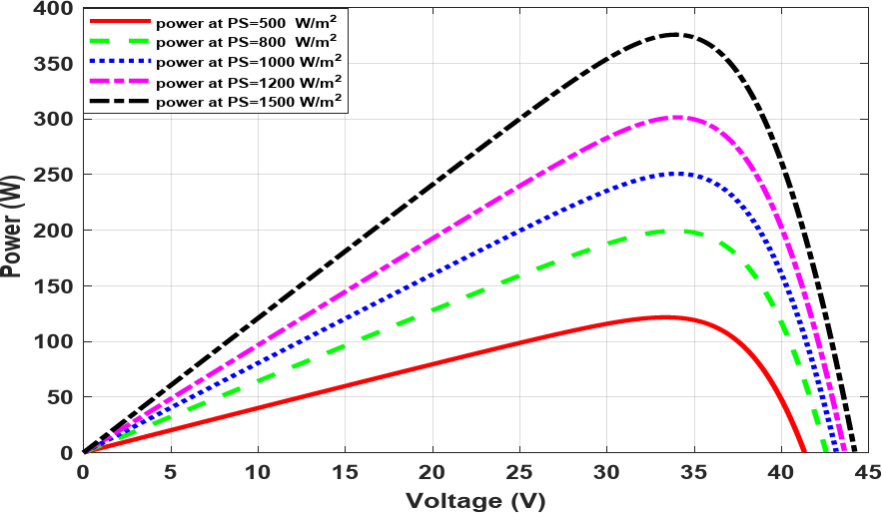
The P-V characteristic at different irradiance.

As the temperature rises, the voltage decreases, as illustrated in Fig. 5, which in turn causes the output power to decrease, as shown in Fig. 6. Conversely, as the irradiance increases, the current increases, as illustrated in Fig. 7, which in turn causes increasing power and, roughly speaking, maintains a constant output voltage, as illustrated in Fig. 8.

### Boost converter

A sizable element of the PV conversion chain is DC-DC converters. These converters change a system’s input voltage to match the intended output voltage. Among them are converters for the buck, boost, buck-boost, and Ćuk. A boost-type converter is typically used as the first stage rather than a transformer to enhance the dual-stage PV’s construction’s broad voltage range or when fewer modules are needed for a given desired output voltage. As seen in Fig. 1, this converter circuit is made up of a power switch (S), diode, capacitor (C), inductor (L), switching controller, and load (R). This architecture can be utilized as an interface link between the PV array and the load to track the PV array’s MPP in addition to adjusting voltage levels. A MOSFET may be used as the switch; it has a variable duty cycle ($\:D$) and an operating frequency ($\:f$) that allow for frequent on/off cycles. The switch’s and diode’s complementary conductivity allows the boost converter to operate in two different modes based on the switch’s position. The switch operates in two modes: the first occurs when it is closed (ON), and the second occurs when it is open (OFF).The relationship between the input and output voltages ($\:{V}_{o}\text{a}\text{n}\text{d}{V}_{pv},\:\text{r}\text{e}\text{s}\text{p}\text{e}\text{c}\text{t}\text{i}\text{v}\text{e}\text{l}\text{y}$) is expressed in the following expression:

5 $$\:{V}_{o}=\frac{1\:}{1-D}{V}_{pv}$$

The following formulas^29^ are used to determine the boost converter parameter values, which are displayed in Table 3.

6 $$\:L=\frac{{V}_{pv}({V}_{o}-{V}_{pv})}{f\:\varDelta\:I\:{V}_{o}}$$

7 $$\:{C}_{2}=\frac{{I}_{o}\left({V}_{o}-{V}_{pv}\right)}{f\:\varDelta\:V\:{V}_{o}}$$

8 $$\:{C}_{1}=\frac{{I}_{o}*D\left(1-D\right)*1000}{f\:\:{V}_{o}}$$

**Table 3:** Parameters of DC-DC boost converter.

No.	Parameters	Value
1	$\:f$	5 kHz
2	$\:C1$	1000 µ f
3	$\:C2$	600 µ f
4	$\:L$	16 mH

PWM and MPPT controllers are the most often utilized types of charge controllers presently. Both methods are often utilized in the off-grid solar industry and are excellent solutions for effectively charging the battery.

#### PWM charge controller

**Fig. 9:**
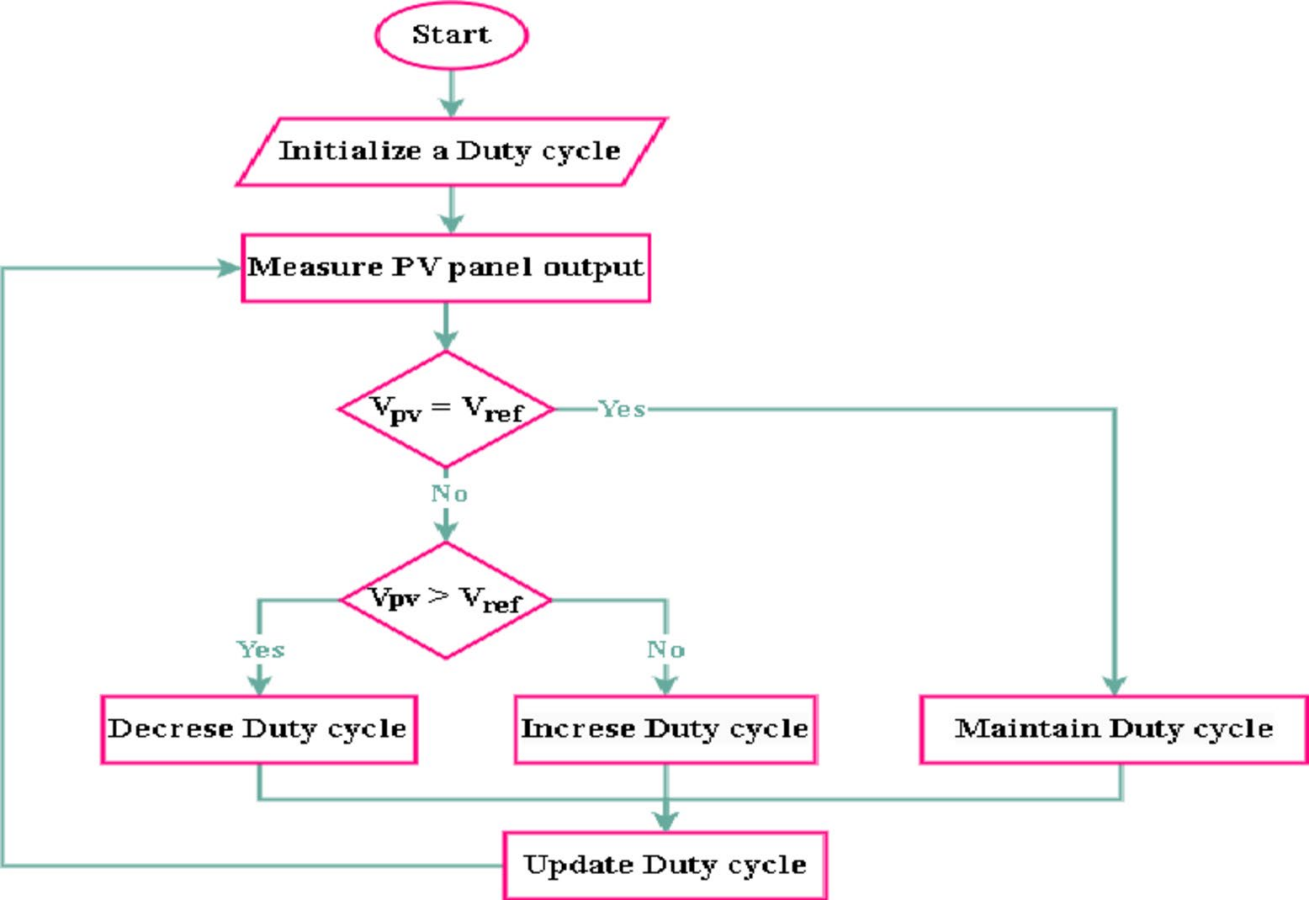
Flowchart of charge controller with PWM technology.

PWM charge controllers are among the most widely used devices for storing solar energy. Here, a switching circuit must be used to process the solar energy input. An oscillator whose pulse width adjusts in response to the output voltage of the PV panel controls this switching circuit. The major goal is to apply a steady voltage to switch the solar system controller power items. PWM controllers operate on the theory that voltage produced by solar cells is signaled by a voltage indicator. Following this measurement, the voltage controller regulates the voltage according to Fig. 9.

#### MPPT charge controller

**Fig. 10:**
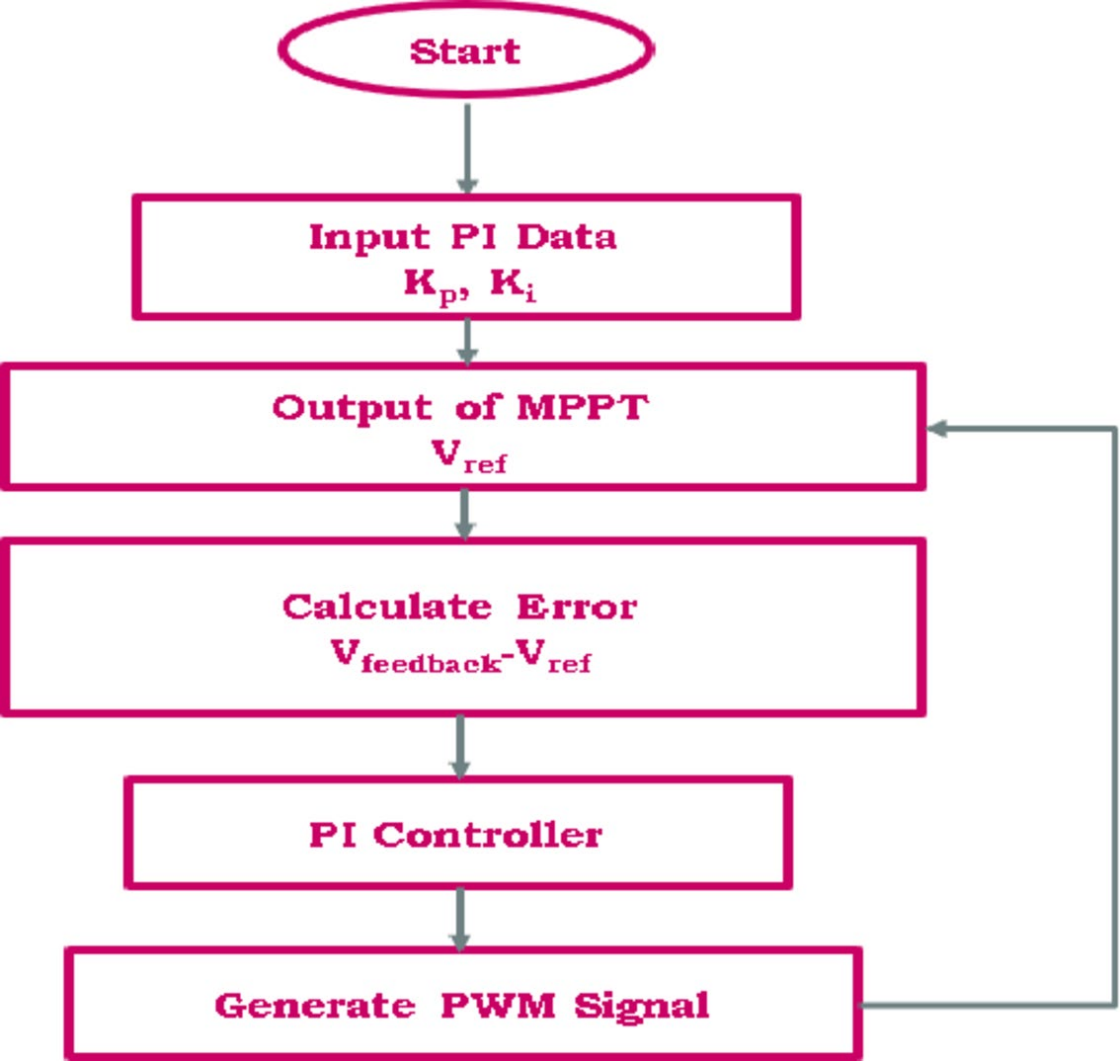
Flowchart of MPPT-PWM charge controller.

MPPT is an essential electronic technology that maximizes the efficiency of solar panels by continuously adjusting their operating voltage and current to extract the maximum available power. Since solar power output varies with irradiance, temperature, and load conditions, maintaining optimal performance requires precise control. Figure 10 illustrates how integrating MPPT with a PI controller significantly enhances the regulation of the PWM (Pulse Width Modulation) signal, ensuring stable and efficient power conversion. The PI controller continuously compares the MPPT voltage ($\:{\varvec{V}}_{\varvec{m}}$) determined by the MPPT technique as a reference voltage with the actual PV module voltage ($\:{V}_{pv}$) and dynamically adjusts the PWM duty cycle to maintain operation at the maximum power point. The proportional component of the PI controller provides an immediate correction to fluctuations, while the integral component eliminates steady-state errors, ensuring long-term stability. This integration is crucial because MPPT alone determines the optimal operating point but requires an efficient control mechanism to maintain it under dynamic conditions. The PI controller refines this process by providing real-time adjustments, improving response speed, and minimizing power losses due to variations in environmental conditions. By working together, MPPT and the PI-controlled PWM system enhance the overall energy conversion efficiency, ensuring maximum power extraction from the PV system. This approach is particularly beneficial for both standalone and grid-connected solar installations, where reliable and efficient power regulation is essential for seamless renewable energy integration.

The MPPT system’s objective is to sample the PV output cell and apply the appropriate resistance to maximize power under all environmental circumstances. MPP is the result of multiplying MPP current ($\:{\varvec{I}}_{\varvec{m}}$) by MPP voltage ($\:{\varvec{V}}_{\varvec{m}}$). Like a DC-DC boost converter, the MPPT solar charge controller converts electricity from a lowerlevel to a higherlevel of voltage. Product $\:\varvec{P}=\varvec{V}\varvec{*}\varvec{I}$ stays fixed if the input voltage is lower than the output voltage because of the inverse relationship; the output current will be below the input. According to this equation, variations in power also imply variations in voltage and current^9^. To get maximum power out of a solar panel, three aspects need to be considered:

i. Irradiance: Modifies the present operational point of a PV panel.
ii. Temperature: Adjusts the operating voltage point of PV panels.
iii. Load: The load acts as a voltage and current reference.

## MPPT controller methods

**Fig. 11:**
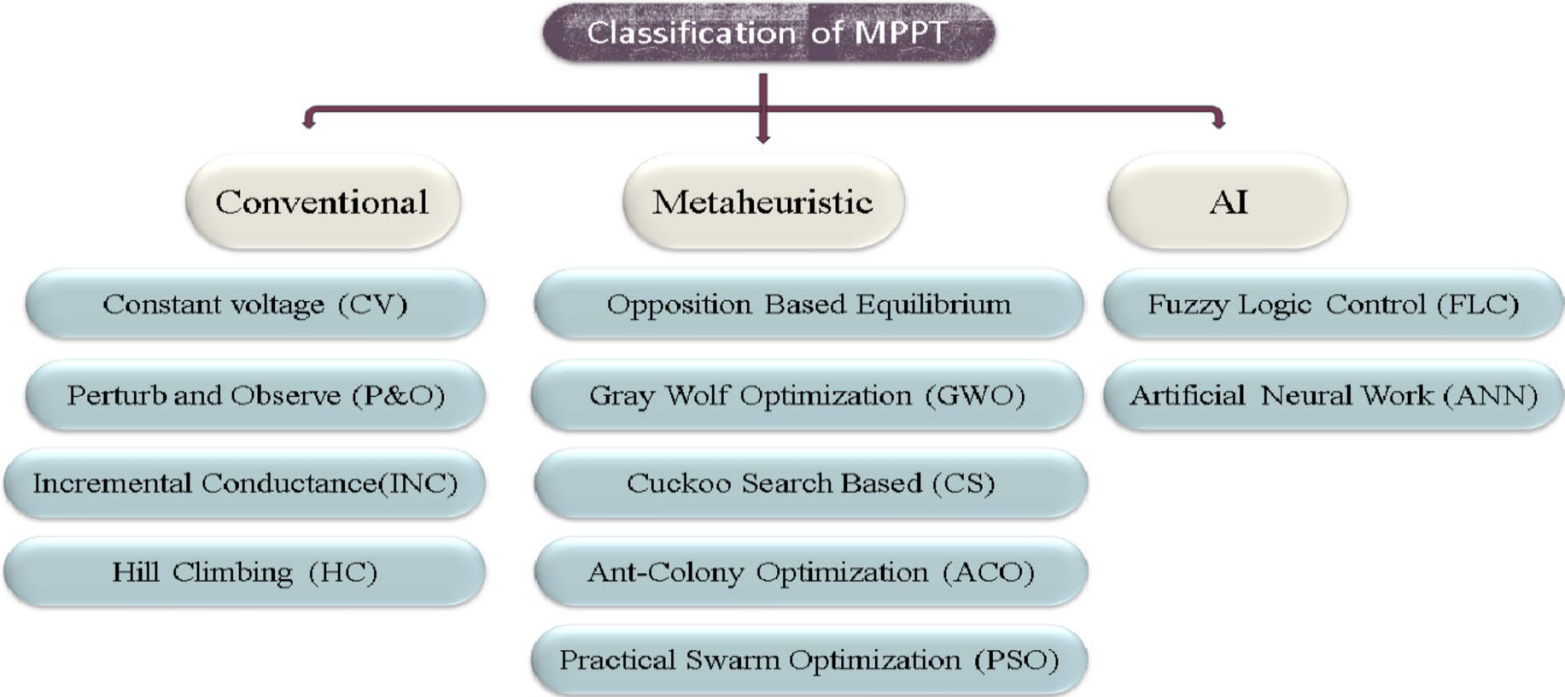
Classification of an MPPT approaches.

Each PV module has a unique MPP depending on the surrounding atmospheric conditions. Therefore, MPPT algorithms are utilized to extract the greatest power from it. Utilizing electronic converters, these algorithms are enforced. Even though these methods improve PV system efficiency under environmental conditions, designers are concerned about tracking global MPP (GMPP). These methods are implemented using MATLAB software. These algorithms sample several PV module parameters regularly and then adjust the duty ratio of the DC converter. By doing this, the PV module perceives a different impedance, allowing for the achievement of maximum power. These MPPT methods are categorized according to Fig. 11^15^. The fundamentals of these methods are thoroughly explained in the sections that follow.

### Conventional MPPT techniques

Typically, traditional MPPT methodologies are employed, such as P&O and INC. The following sectionssummarizethese two methodologies based on their representative equations, characteristics, and measurable variables.

#### Perturb and observe (P&O)

**Fig. 12:**
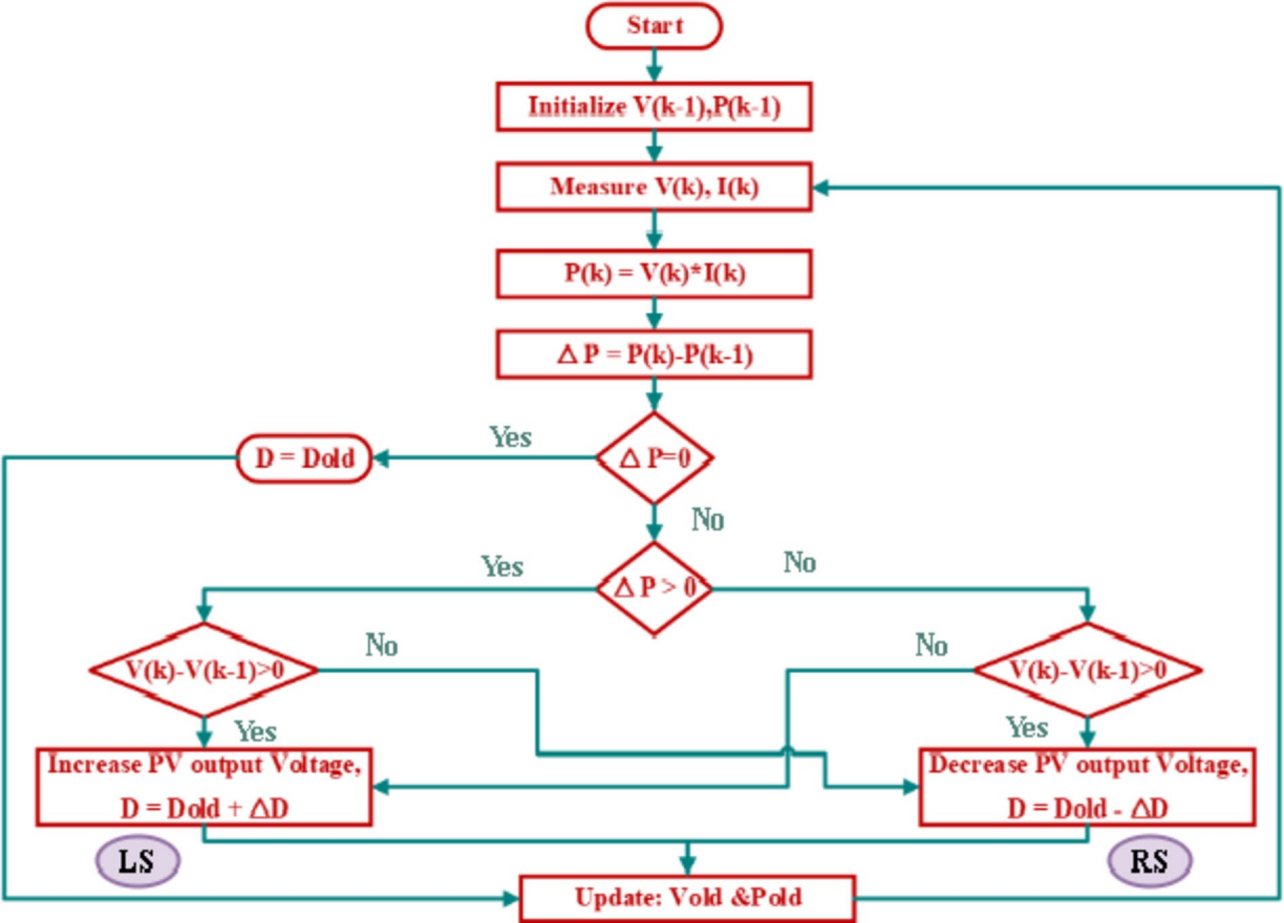
Flowchart of P&O based MPPT for PV system.

The P&O-based MPPT method is prevalent owing to its simplicity, ease of application, minimal sensor needs, and inexpensive prices. Figure 12 depicts the repetitive nature of this MPP tracking technique^8,30^. This approach operates based on small changes in PV array voltage and detects its corresponding influence on power. This is accomplished by adjusting the duty cycle ($\:D$) of the DC-DC converter used in the system. It is possible to calculate the power change using these perturbations. The PV module’s operational point is on the left side of the P-V curve if power is raised by raising the voltage. Conversely, if power decreases as voltage increases, the PV module operating point is located on the right side of the P-V curve. Therefore, to monitor MPP, the direction of the perturbation needs to be such that it converges to a certain destination. This iteration method is then carried out again until MPP is attained. Even though traditional P&O technology performs admirably in steady environmental conditions, it is affected by power ripple, under- and over-shooting, and access time badly since it relies on continuous variation in voltage even when the maximum power is achieved.

#### Incremental conductance (INC)

The INC approach, which is an enhanced form of P&O, permits tracking MPP in a circumstance that changes frequently^21^. This method’s main idea is to calculate the power slope on the PV curve. Instantaneous power is calculated by multiplying instantaneous voltage by instantaneous current, as shown in the equation below:

9 $$\:P=V*I$$

The slope of the P-V curve may be calculated as:

10 $$\:\frac{\partial\:P}{\partial\:V}=\:\frac{\partial\:\left(V*I\right)}{\partial\:V}=I+V\left(\frac{\partial\:I}{\partial\:V}\right)$$

**Fig. 13:**
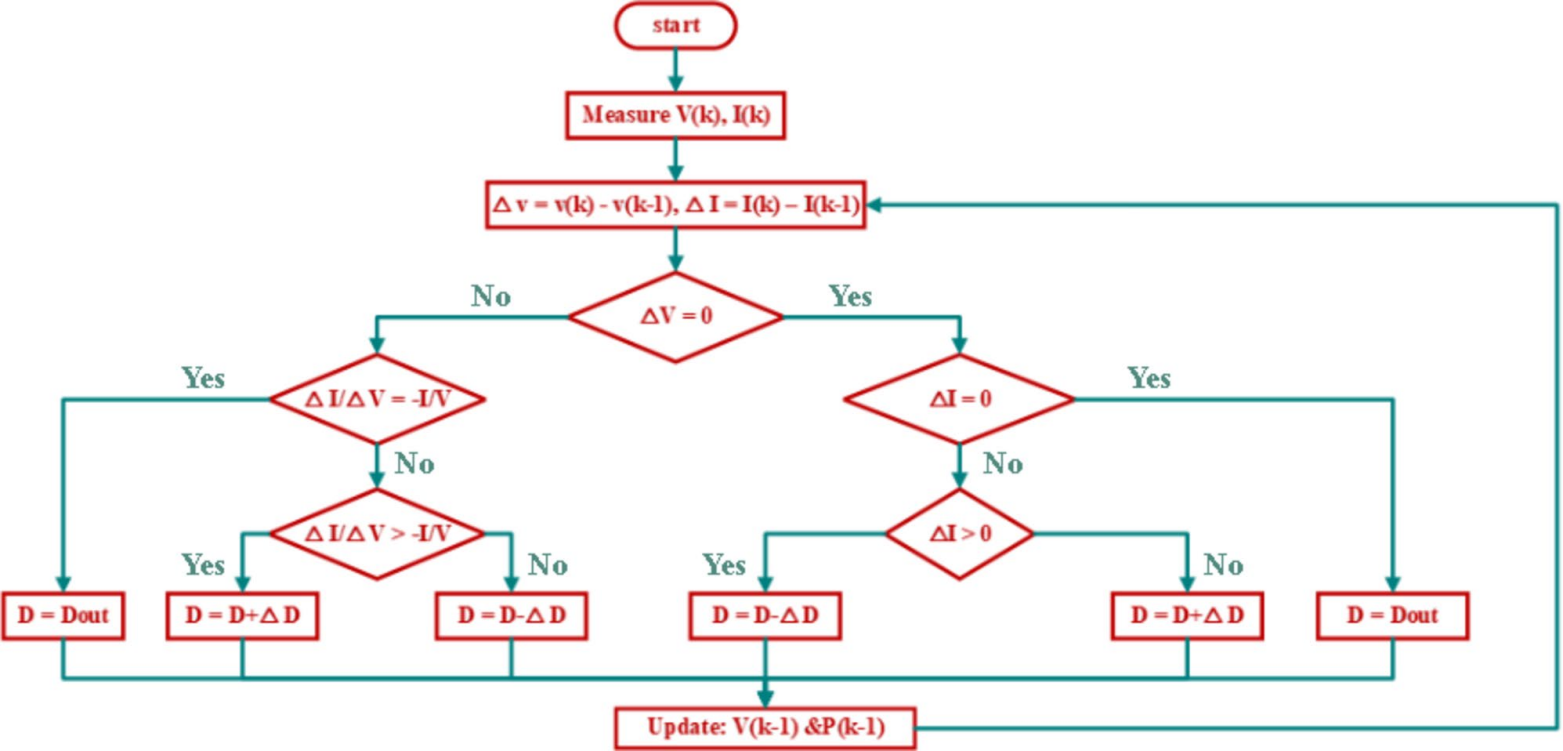
Flowchart of INC based MPPT for PV system.

This technique is based on the proposition that at MPP, the variation in power over the variation in voltage of PV equals zero ($\:dP/dV\:=\:0$) at MPP, a number larger than zero to the left of MPP and smaller than zero to the right. This approach involves measuring and observing current and voltage values. The MPP is computed by calculating the change in voltage ($\:\varDelta\:V$) and current ($\:\varDelta\:I$) and comparing them to reference values. The duty cycle ($\:D$) is then modified by raising or lowering ($\:\varDelta\:D$) and stored in ($\:{D}_{out}$) to achieve the result^31^. This approach identifies whenever the MPPT reaches the MPP, whereas P&O oscillates around it. This provides an advantage against P&O. Additionally, compared to the P&O approach, INC can follow quickly increasing and declining irradiance conditions with greater precision. The drawback of this method is that it is more sophisticated than P&O. Figure 13 provides a flowchart that makes the method easy to understand. To provide a clearer explanation of the working principle of the Incremental Conductance (INC) method, the following is a concise illustration of its underlying logic:

When $$\:\varvec{\varDelta\:}\varvec{V}=0$$:

- If $\:\varDelta\:I\:=\:0$,this condition is achieved when the operating point is at MPP, there is no change in the operating conditions, and the duty cycle remains unchanged *(*$\:D={D}_{out}$*).*
- If $\:\varDelta\:I\:>\:0$, this condition is achieved when the operating point is at the right of MPP for a certain irradiance, so the duty cycle should be decreased $\:(D-\varDelta\:D)$. While applying this duty cycle, the irradiance increased, and the new operating point is at the right of the new MPP for the new irradiance. So, the correct decision is that the duty cycle should decrease $\:(D-\varDelta\:D)$.
- If $\:\varDelta\:I\:<\:0$, this condition is achieved when the operating point is at the left of MPP for a certain irradiance, so the duty cycle should be increased$\:(D+\varDelta\:D$). While applying this duty cycle, the irradiance decreased, and the new operating point is at the left of the new MPP for the new irradiance. So, the correct decision is that the duty cycle should increase $\:(D+\varDelta\:D$).

When $\:\varvec{\varDelta\:}\varvec{V}\ne\:0$The system key check is whether $\:\varDelta\:I/\varDelta\:V\:>\:-I/V$ or $\:\varDelta\:I/\varDelta\:V\:<\:-I/V$.

- If $\:\varDelta\:I/\varDelta\:V\:>\:-I/V$ (left of MPP), the duty cycle should increase ($\:D+\varDelta\:D$).
- If $\:\varDelta\:I/\varDelta\:V\:<\:-I/V$ (right of MPP), the duty cycle should decrease ($\:D-\varDelta\:D$).
- If $\:\varDelta\:I/\varDelta\:V\:=\:-I/V$, if at MPP, $\:D$ remains the same ($\:D={D}_{out}$).

This structured decision-making process ensures that the duty cycle ($\:D$) dynamically adapts to the PV module’s operating conditions, maximizing energy extraction with minimal oscillation. Figure 13 correctly represents these principles, confirming that the incremental conductance method effectively tracks the MPP with improved stability and accuracy.

### Artificial intelligence techniques

There are evolving technologies that try to use technology to mimic the intelligence of humans.The subfields of artificial intelligence (AI) that enable computer systems to learn and adapt entirely novel methods from training data include machine learning (ML) and deep learning.

#### Fuzzy logic control (FLC)

Since fuzzy logic tracks the MPP regardless of the uncertainty of the information sources, their design is considered sensible in the current modern environment where control techniques are evolving to more and higher degrees. Fuzzy controllers don’t need to have an accurate scientific model of the PV system. There are two primary ways in which this FLC is superior to alternative methods:

1. The precise mathematical model of the system is not necessary
2. Controller design is a process that only humans can do.

Expertise from humans is utilized to construct the fuzzy rules, which are one of the three main components. The three stages of a fuzzy approach are generally fuzzification, rule base query tables (fuzzy rules), and defuzzification^17^.

##### Fuzzification

During the fuzzificationprocess, both the error $\:\varvec{E}\left(\varvec{k}\right)$ and change of error $\:\varvec{\varDelta\:}\varvec{E}\left(\varvec{k}\right)$ input variableshave been converted as fuzzy inputs into linguistic variables by use of a membership function (MF), like PB (positive big), PM (positive medium), PS (positive small), ZE (zero), NS (negative small), NM (negative medium), and NB (negative big).The different kinds of membership functions include the Gaussian-specified, trapezoidal, and triangular forms. The membership function of the triangular type is utilized because it has less complexity when splitting values of the low, medium, and high MFs, contrasting with various other MFs. An error ($\:\varvec{E}$) is often one of the inputs to an MPPT-FLC, and a change in error ($\:\varDelta\:\varvec{E}$) is stated in Eqs. (11), (12).

11 $$\:E=\frac{{P}_{in}\left(K\right)-{P}_{in}\left(K-1\right)}{{V}_{in}\left(K\right)-{V}_{in}\left(K-1\right)}\:$$

12 $$\:\varDelta\:E=E\left(K\right)-E\left(K-1\right)$$

**Fig. 14:**
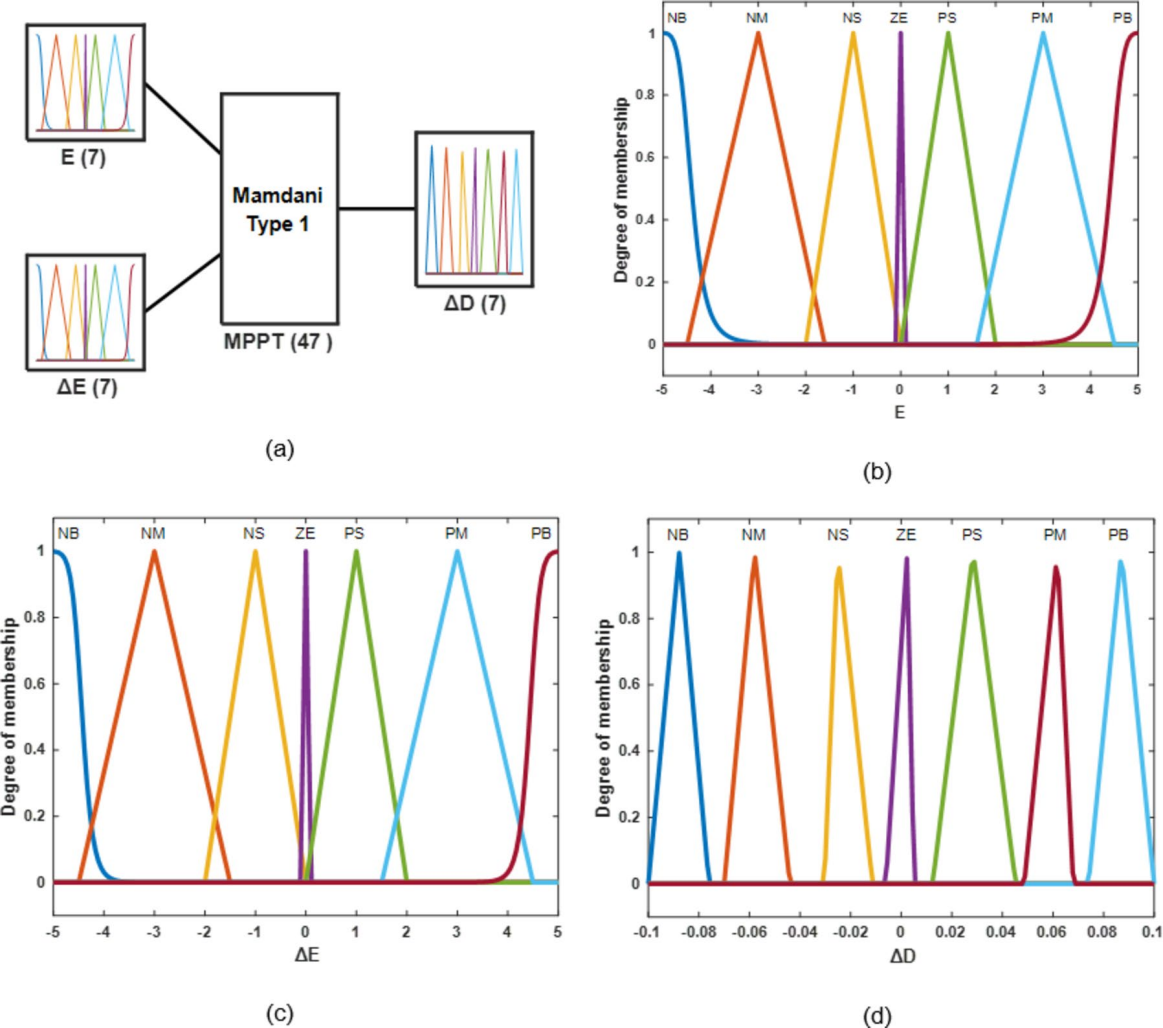
Fuzzy logic designer (**a**) basic structure, and membership functions for (**b**) input error (**c**) input change in error (**d**) output change in duty cycle of fuzzy controller.

Figure 14 shows the basic style of an FLCS and membershipfunctions of $\:E$, $\:\varDelta\:E$ and $\:\varvec{\varDelta\:}D$.

##### Inference engine

The fuzzy inference engine is determined using Mamdani’s technique, which applies a rule to the fuzzy input for defining the fuzzy output. The real input value needs to be fuzzy before the rule is evaluated to obtain a linguistic value that is acceptable. The fuzzy controller rules are listed in Table 4, where every matrix’s entry represents a fuzzy set of inputs ($\:\varvec{E}\left(\varvec{k}\right),\varvec{\varDelta\:}\varvec{E}\left(\varvec{k}\right)$), and the output represents the change in duty ratio ($\:\varvec{\varDelta\:}\varvec{D}$) to the converter. The rules set in Table 4 include 49 fuzzy controller rules. The DC-to-DC converter is monitored using these regulations to ensure that the MPP of the PV module is fulfilled. The control rules are evaluated using a mechanism of inference. The primary purpose of these rules is to relocate the operating point of the PV module closer to the MPP by raising or reducing the DC-to-DC converter’s duty ratio depending on the location of the MPP.

**Table 4:** The proposed rule base correlates $\:\varvec{E}$, $\:\varDelta\:\varvec{E}\:$and controller output $\:\varDelta\:\varvec{D}$.

$\:\varDelta\:E\:$	NB	NM	NS	ZE	PS	PM	PB
E							
NB	NB	NB	NB	NB	NM	NS	ZE
NM	NB	NB	NB	NM	NS	ZE	PS
NS	NB	NB	NM	NS	ZE	PS	PM
ZE	NB	NM	NS	ZE	PS	PM	PB
PS	NM	NS	ZE	PS	PM	PB	PB
PM	NS	ZE	PS	PM	PB	PB	PB
PB	ZE	PS	PM	PB	PB	PB	PB

##### Defuzzification

**Fig. 15:**
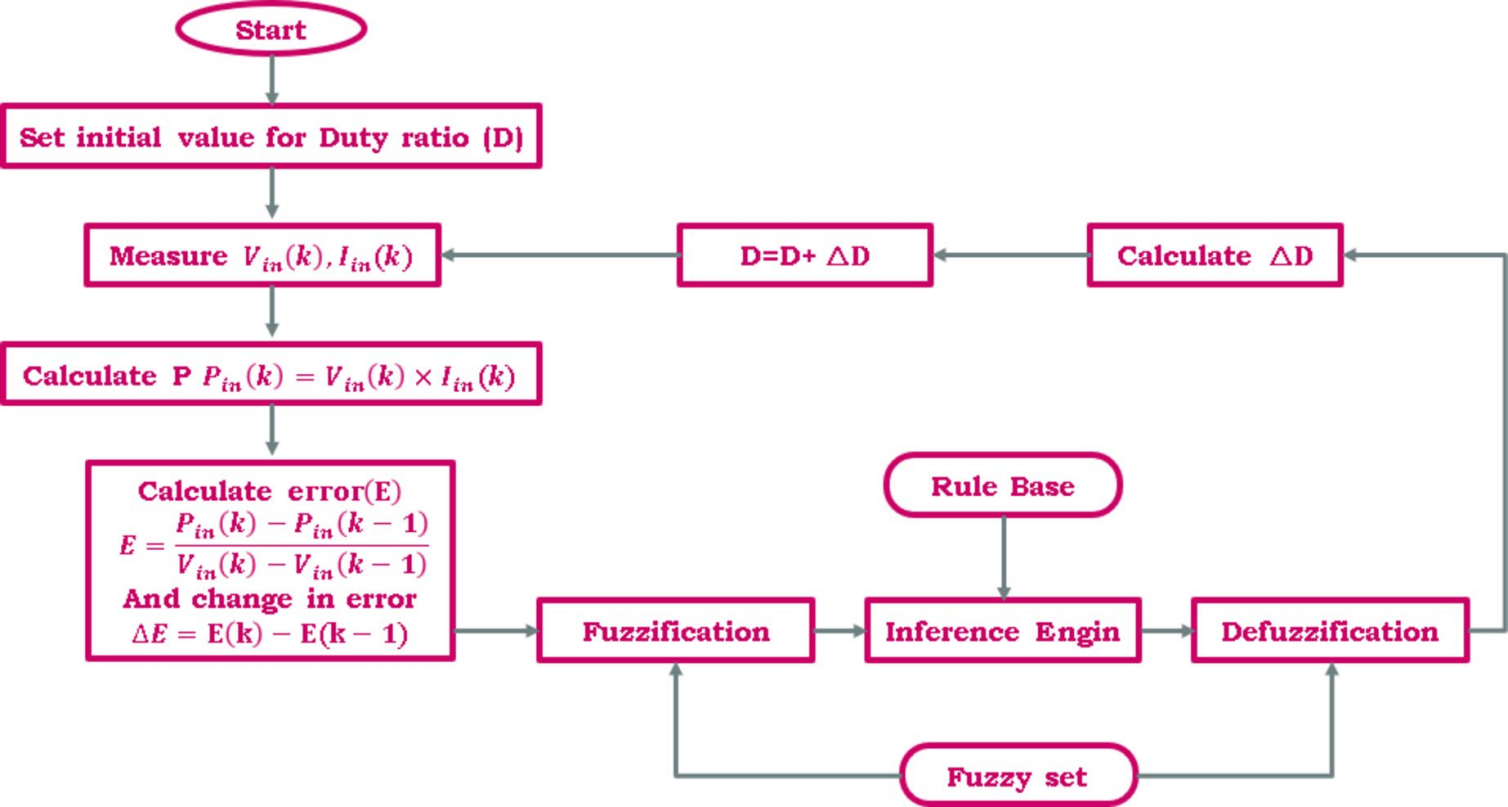
Flow chart of fuzzy logic controller MPPT.

Due to the boost converter’s requirement for a particular $\:\varvec{D}$-control signal upon entry, the fuzzy controller output must first be converted from fuzzy information to crucial information. This transition is known as defuzzification. During the defuzzification step, the output of FLC is converted from a linguistic parameter to a mathematical variable. There are several techniques for defuzzification, such as the max criterion method (MCM), center of area (COA), or center of gravity (COG). The most popular method of defuzzification is to determine the fuzzy combined final set’s COG. According to the relationship of MFs, the final combination in Table 4 uses fuzzy rules control via the maximum aggregation procedure. All the outputs from the fuzzy set of laws are combined to describe the fuzzy set. The following equation is used to find ($\:\varDelta\:\varvec{D}$) the gravity center^31^:

13 $$\:\varDelta\:\varvec{D}=\frac{\sum\:_{\varvec{j}=1}^{\varvec{n}}\varvec{\mu\:}(\varDelta\:{\varvec{D}}_{\varvec{j}})\times\:\varDelta\:{\varvec{D}}_{\varvec{j}}}{\sum\:_{\varvec{j}=1}^{\varvec{n}}\varvec{\mu\:}(\varDelta\:{\varvec{D}}_{\varvec{j}})}$$

where ∆D is the crisp output value, ∆D_j_ is the output MFs’ center of max-min composition, μ(∆D_j_) is the max. of the j_th_MF, and D_j_ is the j_th_ input value. Before being transmitted to the MOSFET’s gate, the output crisp value (∆D) gets added to the duty ratio’s prior value. Figure 15 displays the FLC for the MPPT flowchart.

#### Artificiel neural network (ANN)

This intelligence-driven artificial neural network is among the most effective solutions for addressing highly complicated issues. These ANN applications will necessitate no comprehensive knowledge of the system and mathematical modeling. This is how they may address more intricate issues by accurately delineating the system’s input-output relationships. Artificial Neural Networks (ANN) represent an intelligence-driven advancement in Maximum Power Point Tracking (MPPT) technology, reflecting the biological structure of neurons and the inherent processes of natural learning. In essence, a directed chart with neurons as the nodes and synapses as the edges may be used to describe an ANN^18,30^. Radial basis function networks (RBFNs) can be used to represent one of the simpler function classes. Equation 14 provides the activation function that makes up this output equation.

14 $$\:f\left(x\right)={\sum\:}_{j=1}^{m}{w}_{j}*\:{h}_{j}\left(x\right)$$

Where the incoming signals are denoted by*x*_*1*_; *x*_*2*_;. . .; *x*_*n*_,the weights of the related synapses are represented by.

*w*_*1*_; *w*_*2*_;. . .; *w*_*m*_ and $\:{h}_{j}\left(x\right)$denotes the network’s hidden layers$\:\left({L}_{h}\right)$ overall.

This method’s ability to accurately identify the authentic GMPP depends on both the learning process and the architecture of the ANN. The likelihood of the P-V curve accomplishing the GMPP increases with the quantity of data sets over which it is evaluated. ANNs have neurons that can process information in parallel, unlike earlier approaches. The function used in the hidden layers updates the weights.

**Fig. 16:**
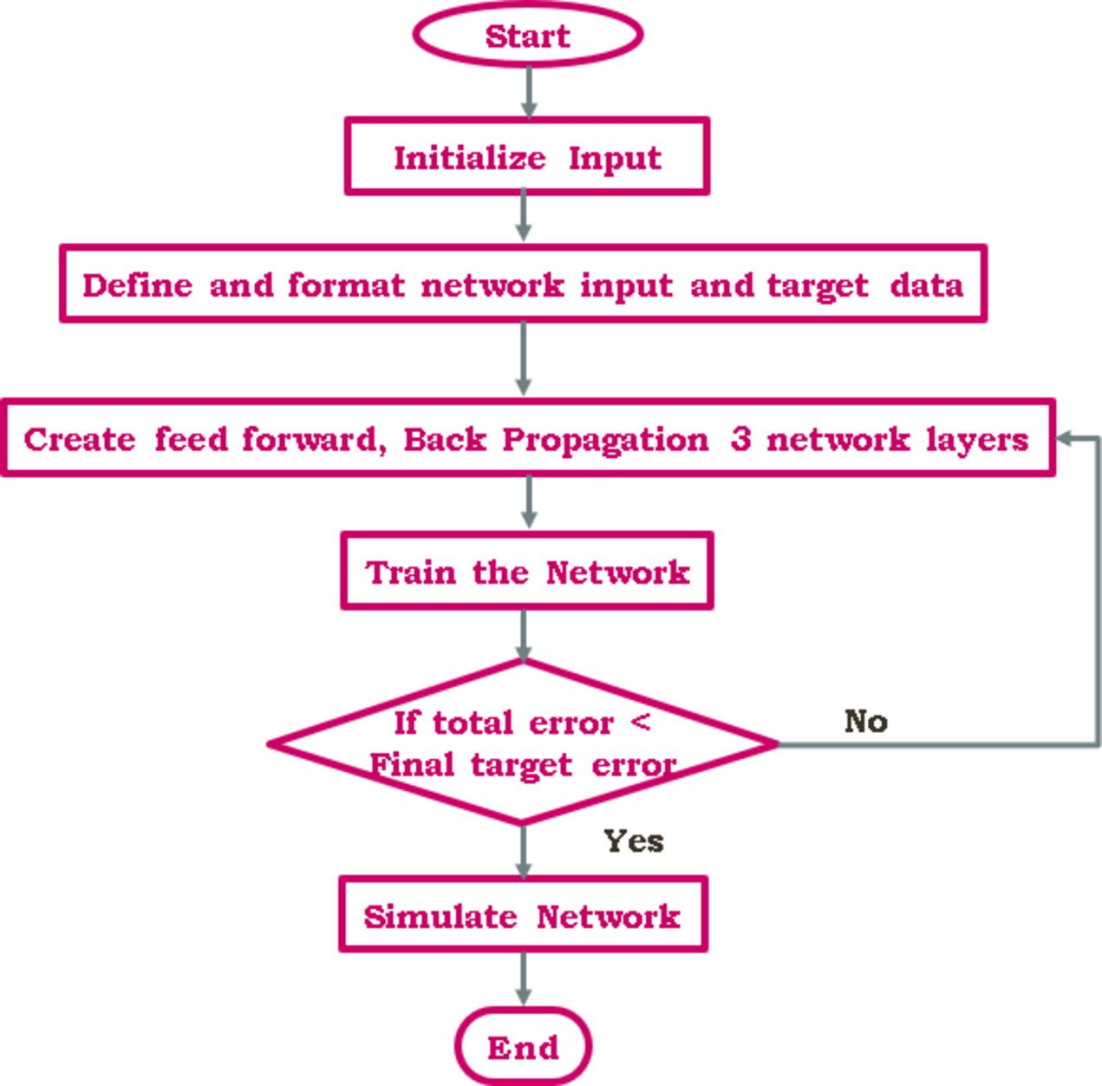
Flow chart of ANN based MPPT.

The MPPT is implemented using a three-layer RBFN-ANN, as seen in Fig. 16. A certain amount of data must be obtained for the neural network’s input and output variables. Both the input and output data come from measurements made experimentally or from simulations using models. There are two input units in the input layer$\:\left({L}_{i}\right)$: the error $\:E\left(k\right)$ and the change in error $\:\varDelta\:E\left(k\right)$. Ten input units make up the hidden layer, whereas the $\:\left({L}_{o}\right)$ only has the duty cycle change ($\:\varDelta\:D)$unit. To get the electronic converter operating close to or at the MPP, the output NN ($\:\varDelta\:D)$ is added to the duty cycle’s prior value. The system will function better if the weight of the linkages is enhanced and the learning settings are adjusted. As a result, weights for neurons in various layers are acquired. Several methods exist for training AIs. The ANN is trained in this study using the error backpropagation approach. Ten tansig neurons are the ideal quantity of neurons for the first layer, while one purelin neuron is present in the second layer.

### Meta-heuristic

Some of the limitations of conventional techniques include local optima convergence, single-based solutions, and challenges with unknown search spaces. To overcome these limitations, several metaheuristics have been developed. Metaheuristic methods have much better performance than conventional methods. Thus, we can create heuristic and/or meta-heuristic algorithms that are inspired by nature and learn from them how to solve problems successfully. As such, they are referred to as bioinspired or simply biology-inspired. Any meta-heuristic algorithm should have two main parts: randomization and the selection of the best solutions. While randomization prevents solutions from becoming stuck at local optima and increases solution diversity, best-fit selection guarantees that the solutions will converge optimally. A successful amalgamation of these two constituents will typically guarantee the attainment of global optimal.

#### Practical swarm optimization (PSO)

The PSO method, a metaheuristic algorithm, imitates how fish or animals might behave when they’re looking for food. Particles use this method to move around the search space randomly. The information about the greatest food is shared by a particle that discovers it with the others, directing them to the best area to procure food^32^. This technique may be used in solar panels as an MPPT approach. Particles must initially acquire duty cycle information and then estimate their related output power. The particle exhibiting the greatest output of power is used as a reference for other particles, which modify their motion to arrive at this optimum location. Throughout its trip, every particle maintains a record of its “personal best,” or optimal solution. Local or individual experience ($\:{P}_{best}$), together with the experiences of other particles or the whole of experiences ($\:{G}_{best}$), and the particle’s decision to explore the next location in the multidimensional search space, motivate the algorithm. As a result, the PSO algorithm combines local and global search techniques.

**Fig. 17:**
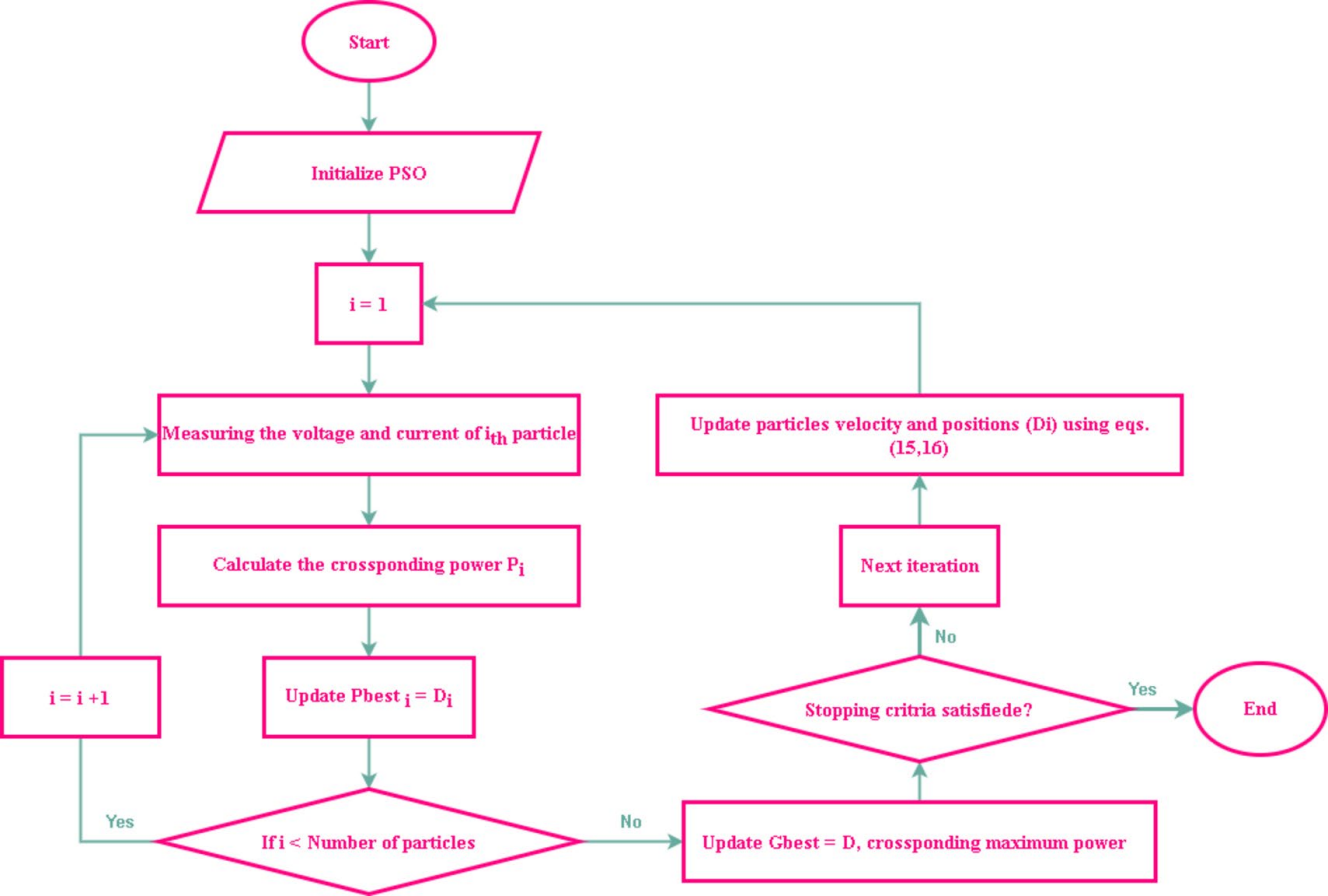
Flow chart of PSO algorithm based MPPT.

The PSO method starts by randomly selecting the initial particle position and then upgrading to the new position to look for the optimal value. Each particle’s location will be updated for every iteration according to the best solution for the best local value ($\:{P}_{best}$) and the best solution based on the global population ($\:{G}_{best}$). After that, Eqs. (15, 16) will be used to update the particle’s location and velocity. Furthermore, the PSO algorithm flowchart is explained in Fig. 17.

15 $$\:{v}_{i}^{k+1}=w{v}_{i}^{k}+{c}_{1}{r}_{1}\left({P}_{best,i}^{k}-{x}_{i}^{k}\right)+{c}_{2}{r}_{2}\left({G}_{best}-{x}_{i}^{k}\right)$$

16 $$\:{x}_{i}^{k+1}={x}_{i}^{k}+{v}_{i}^{k+1}$$

where,$\:w$is the inertia weight that maintains a balance between the local and global search, $\:{c}_{1}$ and $\:{c}_{2}$ are the acceleration coefficients, $\:{r}_{1}$and$\:{r}_{2}$are random values ranging between [0, 1], the $\:{i}_{th}$ particle’s personal best position is denoted by$\:{P}_{best,i}^{k}$,and $\:{k}_{th}$iteration, $\:{G}_{best}$is the particle population’s overall global best position, $\:{v}_{i}^{k}$is the velocity of $\:{i}_{th}$particle and $\:{k}_{th}$iteration, and $\:{x}_{i}^{k}$ is the position of $\:{i}_{th}$particle and $\:{k}_{th}$iteration.

While velocity indicates the step size (perturbation) in the current duty cycle, position is recognized as the actual duty cycle. The current duty cycle perturbation is contingent upon $\:{P}_{best,i}^{k}$and $\:{G}_{best}$. PSO might thus be viewed as an adaptable version of conventional methodologies. In the last case, the duty cycle perturbation is fixed, whereas in PSO, it fluctuates based on particle location. With an appropriate choice of controlling parameters, an appropriate MPPT controller employing PSO may be simply developed.

## Problem formulation

Solar panels have a non-linear P-V characteristic, which means that for each level of sunlight and temperature, there is a specific point known as MPP where the system can generate the maximum possible power. Identifying and maintaining operations at this point is challenging due to fluctuations in environmental conditions. Traditional methods often fail to adapt quickly to these changes, resulting in suboptimal power output. Hence, there is a need for efficient and adaptive MPPT algorithms that can reliably track the MPP under varying conditions.

### Objective function

In this paper, the objective function to be maximized is formulated as shown in Eq. (17) based on the V-I characteristics of the PV panel:

17 $$\:\varvec{M}\varvec{a}\varvec{x}\varvec{i}\varvec{m}\varvec{i}\varvec{z}\varvec{e}\:{\varvec{P}}_{\varvec{p}\varvec{v}}\left(\varvec{D}\right)={\varvec{I}}_{\varvec{p}\varvec{v}}\varvec{*}{\varvec{V}}_{\varvec{p}\varvec{v}}\left(\varvec{D}\right)$$

Subject to the constraint:

18 $$\:{\varvec{D}}_{\varvec{m}\varvec{i}\varvec{n}}\:\le\:\:\varvec{D}\:\le\:\:{\varvec{D}}_{\varvec{m}\varvec{a}\varvec{x}}$$

where$\:{\varvec{P}}_{\varvec{p}\varvec{v}}$is the PV output power,$\:{\varvec{I}}_{\varvec{p}\varvec{v}}$ and $\:{\varvec{V}}_{\varvec{p}\varvec{v}}$are the output current and voltage respectively, $\:{\varvec{D}}_{\varvec{m}\varvec{i}\varvec{n}}$and$\:{\varvec{D}}_{\varvec{m}\varvec{a}\varvec{x}}$represent minimum and maximum duty cycle values, respectively.

## Multi-criteria decision making (MCDM)

MCDM is a cutting-edge area of research that supports analysts and decision-makers by offering them a variety of scientific approaches appropriate for complicated decision issues^33,34^. It can also be defined as the process of choosing, rating, or giving a set of options a higher priority based on independent, competing features or criteria and then identifying the best option out of several possibilities. Calculating the weights of the criteria that are significant for alternatives is a prerequisite for applying MCDM. Subjective weighting methods, such as the Delphi method, the analytic hierarchy process (AHP), the pairwise comparison approach, SIMOS, updated SIMOS, SWING, and the simple multi-attribute rating technique (SMART), etc., are used in the methodology of estimating criterion weights based on the opinions of decision-makers. These methods are affected by the understanding and expertise of decision-makers in the issue’s field and related fields. However, according to solving mathematical models, the weights are established using objective weighting methods (such as mean weight (MW), statistical variance procedure, standard deviation (SD), EW, criteria importance through inter-criteria correlation (CRITIC), etc.) without taking the decision-maker’s preferences into account. Once the weights of the criteria have been determined, various MCDM systems (TOPSIS, WAPSAS, VIKOR, PROMETHE, etc.) concentrate on ranking options when there are conflicting criteria^35^.

The present investigation has opted to employ CRITIC, MW, and AHP as its weighting techniques. These methods primarily rely on objective weights that are derived through the quantification of intrinsic information about each criterion, as well as subjective weights that are shaped by the decision-makers background knowledge and expertise regarding this issue. Since VIKOR ranks among the most often used MCDM techniques for complex systems, it has been chosen for the ranking. In cases where there are competing criteria, it rates and establishes which option is the best and worst. Additionally, the VIKOR technique compares the proximity degree with the optimum solution to get a compromise ranking. The VIKOR strategy’s important characteristic is its ability to minimize individual regret while optimizing collective benefits, hence facilitating the decision-maker’s acceptance of the outcome^36^. The subsequent sections will provide a detailed explanation of these techniques.

### The CRITIC method

Through this procedure, objective weights for criteria are determined through the following steps to obtain the $\:{\varvec{j}}_{\varvec{t}\varvec{h}}$ criteria weights^35^:

**Table:** 

1. Create the decision matrix $~(X = \left[ {x_{{ij}} } \right]_{{m*n}} ,~$ where i = 1, 2 …, m and j = 1, 2, …, n).
2. Standardize the choice matrix,$\:{\varvec{r}}_{\varvec{i}\varvec{j}}=\frac{{\varvec{x}}_{\varvec{i}\varvec{j}}-{\varvec{x}}_{\varvec{j}}^{\varvec{w}\varvec{o}\varvec{r}\varvec{s}\varvec{t}}}{{\varvec{x}}_{\varvec{j}}^{\varvec{b}\varvec{e}\varvec{s}\varvec{t}}-{\varvec{x}}_{\varvec{j}}^{\varvec{w}\varvec{o}\varvec{r}\varvec{s}\varvec{t}}},\:$where,$\:{\varvec{r}}_{\varvec{i}\varvec{j}}$is the standardized score value of$\:{\varvec{i}}^{\varvec{t}\varvec{h}}$option on the$\:{\varvec{j}}^{\varvec{t}\varvec{h}}$factor. In this case,$\:{\varvec{x}}_{\varvec{i}\varvec{j}}$ represents the actual value of $\:\varvec{i}$ alternative for criteria $\:\varvec{j}$, $\:{\varvec{x}}_{\varvec{j}}^{\varvec{b}\varvec{e}\varvec{s}\varvec{t}}$ denotes criterion $\:\varvec{j}$ ‘s best value, and $\:{\varvec{x}}_{\varvec{j}}^{\varvec{w}\varvec{o}\varvec{r}\varvec{s}\varvec{t}}$ denotes criterion $\:\varvec{j}$ ‘s worst value.
3. Compute the standard deviation, $\:{\varvec{\sigma\:}}_{\varvec{j}}$, for each factor $\:{\varvec{\sigma\:}}_{\varvec{j}}=\sqrt{\frac{\sum\:_{\varvec{i}=1}^{\varvec{m}}{\left({\varvec{x}}_{\varvec{i}}-\stackrel{-}{{\varvec{x}}_{\varvec{i}}}\right)}^{2}}{\varvec{N}}}$, where, $\:\stackrel{-}{{\varvec{x}}_{\varvec{i}}}$represents the mean of each factor, and$\:\:\:\varvec{N}\:$is the quantity of values of each criterion.
4. Determine the linear correlation coefficient between the vectors$\:{x}_{i}$ and $\:{x}_{j}$ by element $\:{\rho\:}_{jk}=\frac{\sum\:_{i=1}^{m}\left({r}_{ij}-\stackrel{-}{{r}_{j}}\right)\left({r}_{ik}-\stackrel{-}{{r}_{k}}\right)}{\sqrt{\sum\:_{i=1}^{m}{\left({r}_{ij}-\stackrel{-}{{r}_{j}}\right)}^{2}\sum\:_{i=1}^{m}{\left({r}_{ik}-\stackrel{-}{{r}_{k}}\right)}^{2}}}$, where: $\:{r}_{jk}$ is the correlation coefficient between the $\:{j}_{th}$ and $\:{k}_{th}$ criteria.
5. Measure the conflict brought about by criterion$\:\:j$concerning the choice scenario established by the other criteria$\:\sum\:_{k=1}^{n}\left({1-\rho\:}_{jk}\right)$.
6. Evaluate the amount of data regarding each criterion, $\:{C}_{j}={\sigma\:}_{j}\text{*}\sum\:_{k=1}^{n}\left({1-\rho\:}_{jk}\right)$.
7. Determine the objective weights, $\:{W}_{j}=\frac{{C}_{j}}{\sum\:_{k=1}^{n}{C}_{k}}$.

### The AHP method

**Fig. 18:**
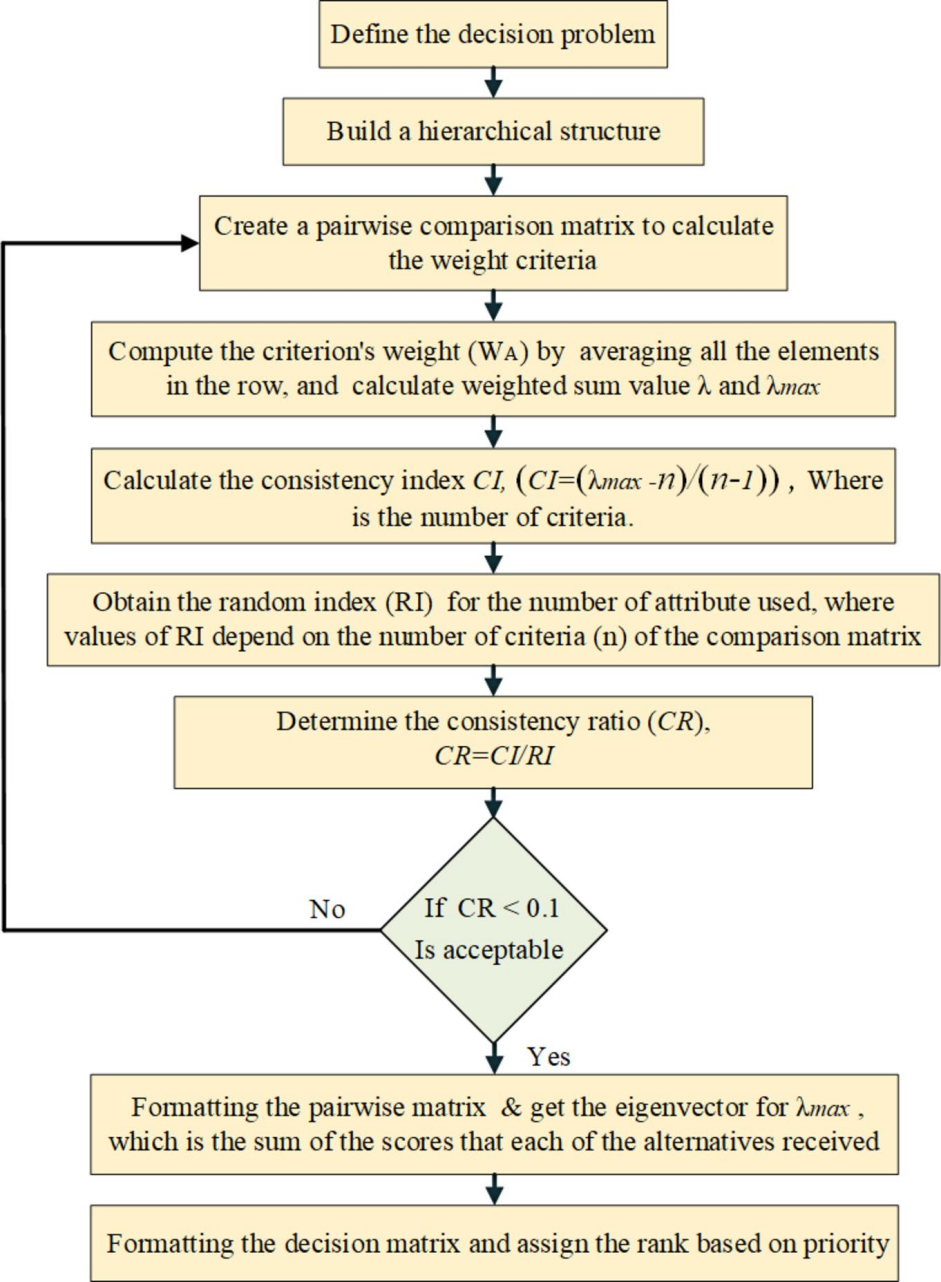
Flow chart of the AHP approach.

The AHP is a method for subjective weight calculation. It is an MCDM technique that can deal with problems of decision-making. The AHP implementation steps are shown in Fig. 18^37^:

### AHP-CRITIC combined weight

Although CRITIC weights illustrate useful and effective information, they heavily depend on objective data and don’t take into consideration the expert’s experience and knowledge wealth in deciding, which may be incompatible with the reality and understanding of the problem situation. Therefore, it is necessary to combine the objectivity of the CRITIC weight and the subjectivity of the AHP method to guarantee effectiveness and reliability for the obtained weights. Integrating the two methodologies aims to gain weight combined between the experts’ experience and knowledge and the objective variability of the evaluation data. After the calculation of the AHP subjective weight vector $\:{W}_{A}$ and the CRITIC objective weight$\:{W}_{C}$, we can get a hybrid weight $\:{W}_{h}$for *n* criteria using the following equation^38^:

19 $$\:{W}_{h}=\frac{{W}_{Aj}{W}_{Cj}}{\sum\:_{j=1}^{n}{W}_{Aj}{W}_{Cj}}$$

where$$\:j$$ = 1, 2, 3, …., $$\:n$$

### VIKOR method

**Fig. 19:**
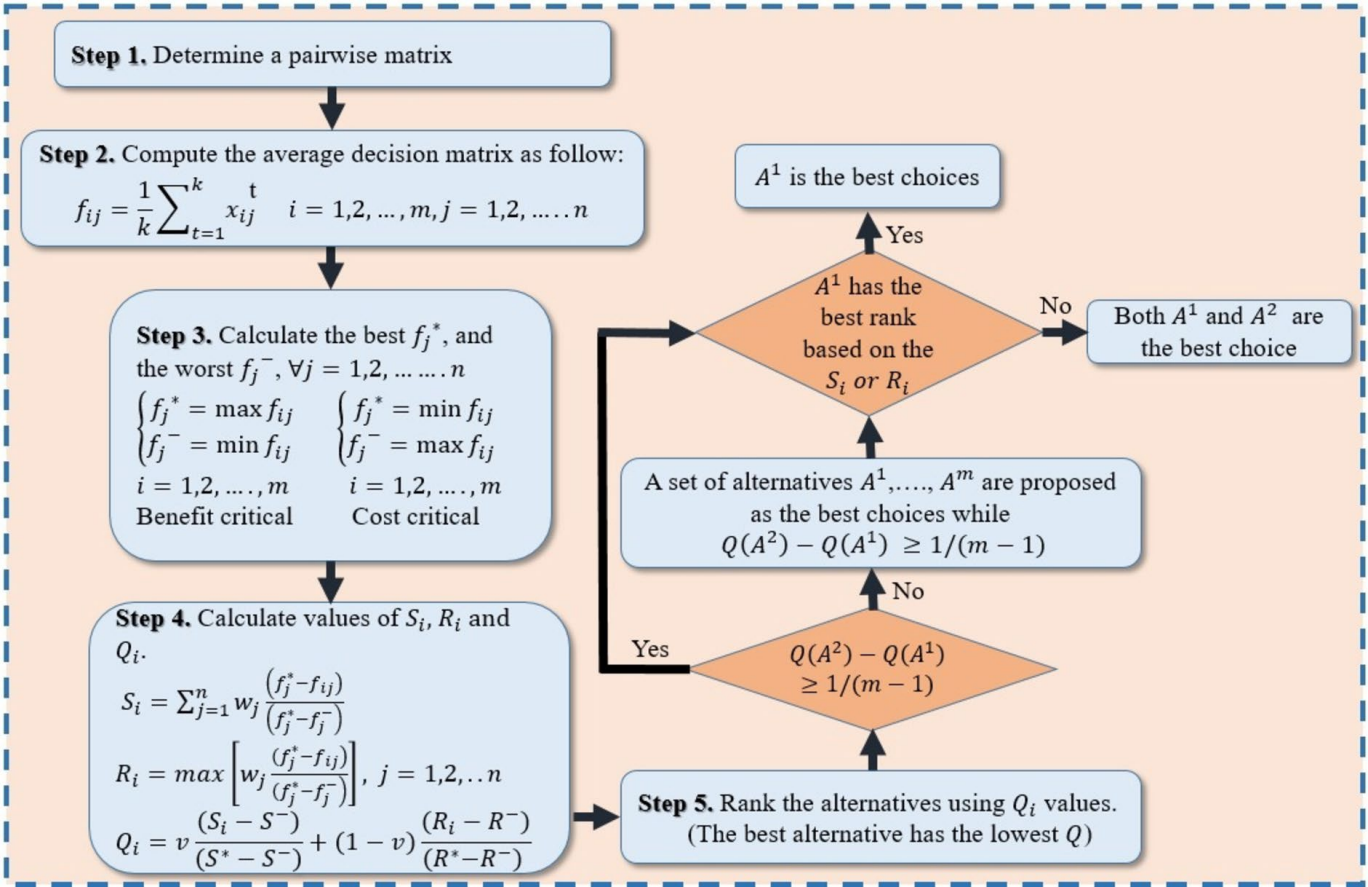
VIKOR ranking steps.

VIKOR is among the appropriate MCDM approaches for tackling optimization issues of complicated systems with multiple criteria, ranking various alternatives, and identifying the best among them^39^. It establishes a compromise ranking list and intervals of weight stability for the compromise solution that is arrived at^40^. Figure 19 clarifies the steps that comprise the VIKOR compromise ranking algorithm.

## Results & discussion

**Fig. 20:**
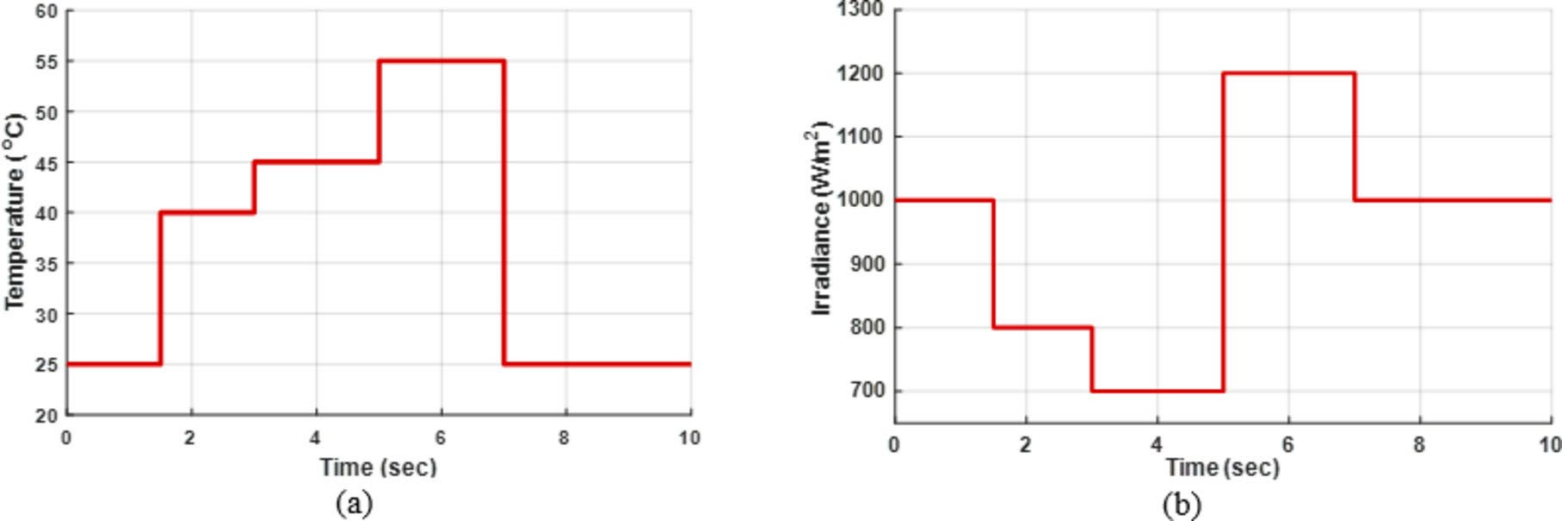
(**a**) Changing of temperature, (**b**) Changing of irradiance.

To demonstrate the importance of MPPT technology, different methods have been applied and compared with the PWM technique. All proposed methods have been run on MATLAB software 2019a, a 3.20 GHz AMD Ryzen 7 5800 H PC with 16 GB RAM. The subsequent sections feature three case studies:

- Case 1: Changing of temperature (Fig. 20a) at a constant irradiance of 1000 W/m^2^.
- Case 2: Changing of irradiance (Fig. 20b) at a constant temperature of25^°^C.
- Case 3: Simultaneously changing temperature and irradiance (Fig. 20).

All three previous cases have been studied using seven techniques.

- TEC. 1: PWM.
- TEC. 2: PWM with PI controller.
- TEC. 3: P&O.
- TEC. 4: INC.
- TEC. 5: PSO.
- TEC. 6: ANN.
- TEC. 7: FLC.

After implementing all MPPT techniques, the MCDM method was applied for ranking and to select the best technique. The parameter setting of the chosen algorithms is shown in Table 5.

**Table 5:** The control parameters of the proposed optimization techniques and operative restrictions.

Algorithm	Parameters	Value
PWM with PI controller	$\:{V}_{ref}^{finite}$	34.04
	$\:{V}_{ref}^{max}$	43.2
	$\:{V}_{ref}^{min}$	0
	$\:\varDelta\:{V}_{ref}$	0.0001
	$\:{K}_{p}$	0.001
	$\:{K}_{i}$	0.01
P&O	$\:{D}_{finite}$	0.8234
	$\:{D}_{max}$	0.95
INC	$\:{D}_{min}$	0.1
	$\:\varDelta\:D$	0.0001
PSO	$\:{c}_{1}$	1.05
	$\:{c}_{2}$	1.05
	$\:W$	0.8
	$\:{N}_{P}$	4
ANN	neurons in $\:{L}_{i}$	2
	neurons in $\:{L}_{o}$	1
	Neurons in $\:{L}_{h}$	10
	Training	1000
FLC	Input 1 ($\:E$)	7 MF
	Input 2 ($\:\varDelta\:E$)	7 MF
	Output ($\:\varDelta\:D$)	7 MF
	Rules	49

To evaluate the proposed methods and analyze their performance, voltage, current, and power are monitored while systematically varying temperature and irradiance, as illustrated in Fig. 20.

### Case 1: changing in temperature

**Fig. 21:**
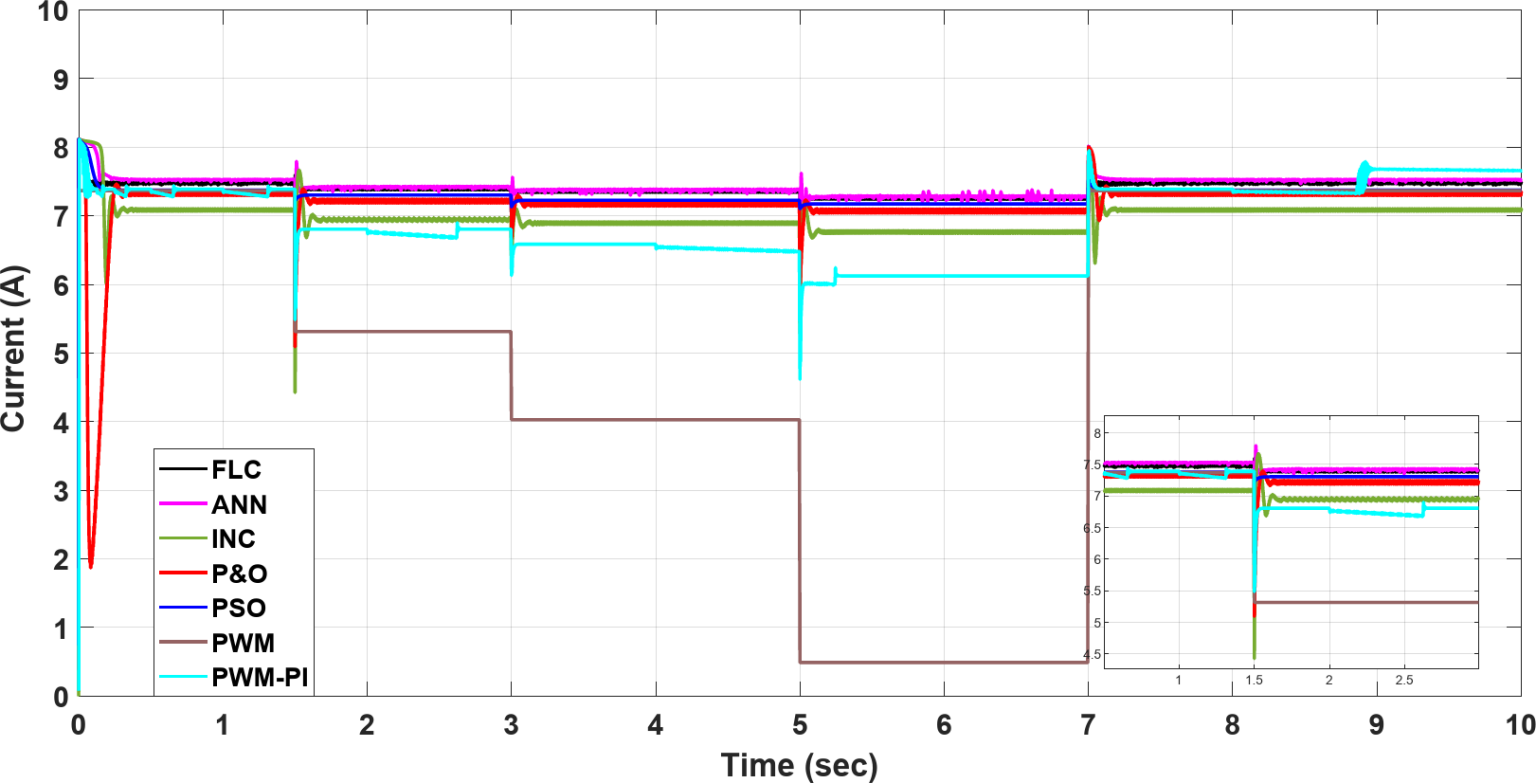
The current profile vis temperature changing.

In this case, the current remains almost constant at around 7.4 A, while voltage and power are inversely proportional to temperature. More precisely, in the situation of PWM, the current is highly sensitive to changes in temperature, leading to a minimal value of 0.5 A at a temperature degree of 55 °C. However, in contrast to other methods, the current of the PWM-PI controller is less affected by temperature changes and reaches 6.2 A at the same temperature degree. For the P&O and INC methods, there is a significant initial drop to 2 A at 1.5 s, followed by slight reductions to 4.5 A at 1.5 s, 6.3 A at 3 s, 5.2 A at 5 s, and 6.3 A at 7 s for the P&O method, which means that this method is very affected by the changing temperature. INC established 6 A as the largest drop at the initial, followed by a slight change around 0.5 A against every temperature change, which means this method is more stable than P&O and achieves moderate performance for varying conditions. Also, PSO, FLC, and ANN methods show a low undershoot of 0.2 A and a ripple of 0.01 A. On the other hand, the PSO, ANN, and FLC are more stable methods because they can maintain the output current at an optimal stable level of nearly 7.4 A and keep it almost constant, as shown in Fig. 21.

**Fig. 22:**
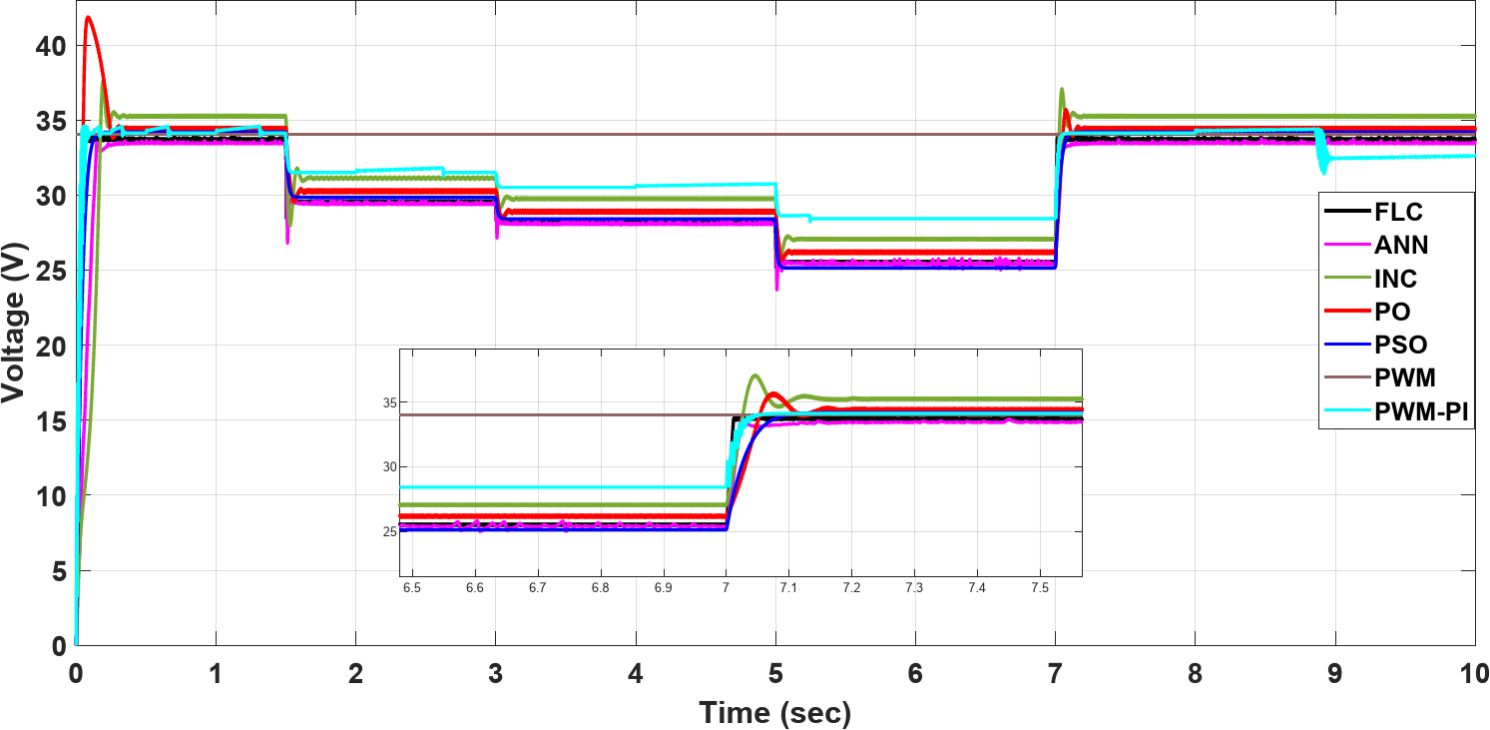
The voltage profile vis temperature changing.

The voltage of the PWM technique remains constant at 34 V throughout all time durations. In contrast, the PWM-PI controller dynamically adjusts its voltage to compensate for current drops caused by temperature changes, aiming to maintain optimal power output. The voltages of other techniques, however, fluctuate with temperature. Notably, the P&O and INC methods exhibit the highest voltage surges at the start, with 8 V and 4 V increases, respectively. FLC and PSO methods achieve the optimal voltage without shooting and minimal ripple. On the other hand, the FLC is the more stable method because it converges faster than other methods with a settling time of 0.01 s. Finally, the inverse relationship between voltage and temperature can be demonstrated by comparing Fig. 20a with Fig. 22, which demonstrates the success of the proposed algorithms with the variation in performance as shown in Fig. 22.

**Fig. 23:**
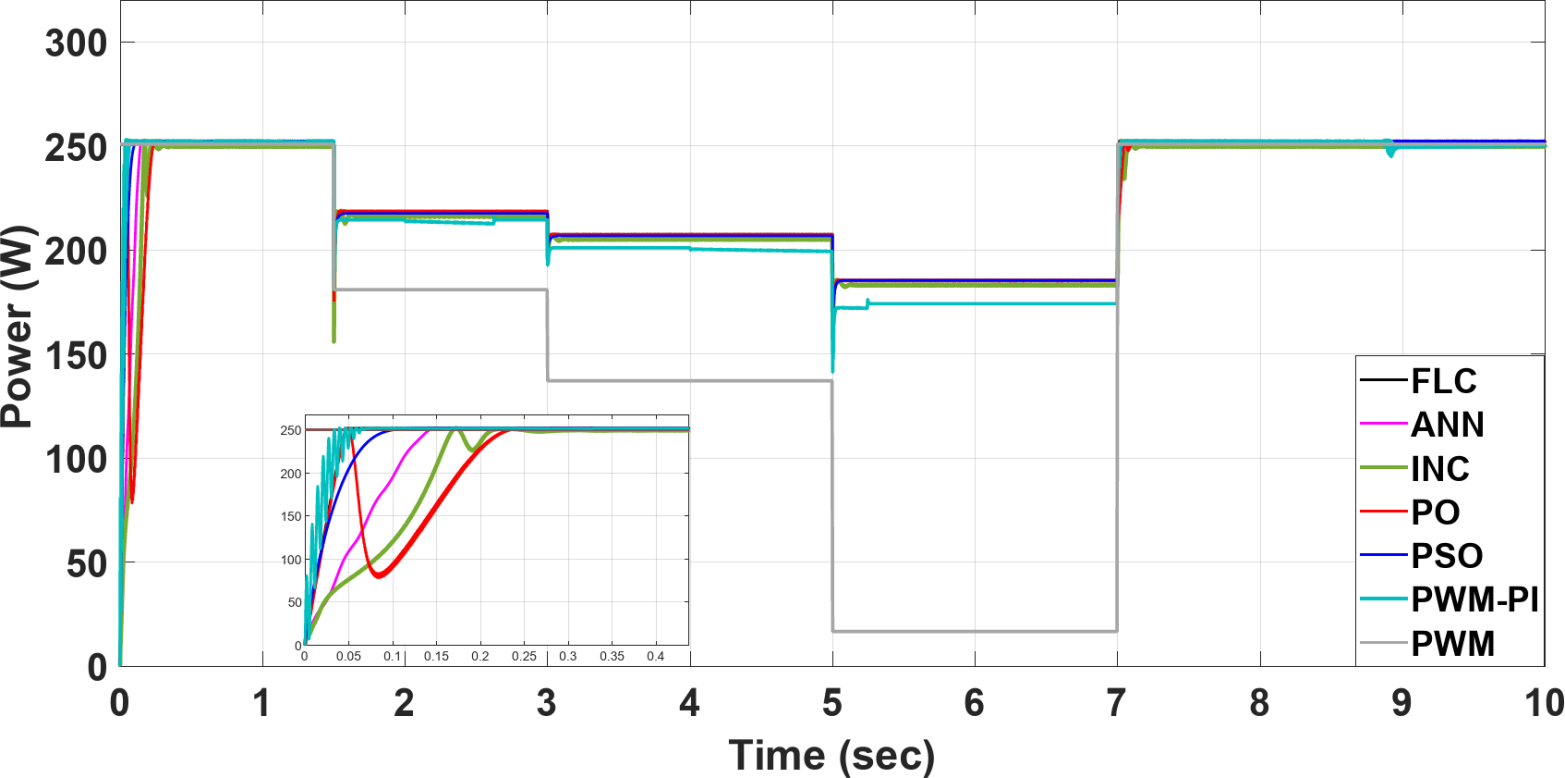
The extracted power response to temperature changing.

The output power of the PWM technique is optimal at nominal temperature but decreases significantly with rising temperatures, dropping to 20 W. Conversely, the PWM-PI controller achieves the lowest output power among the controlled techniques, reaching 200 W at 45 °C. The PSO, ANN, and FLC methods demonstrate the highest output power values, approaching the optimal level of 185 W around 55 °C. The P&O and INC methods exhibit the highest variations in current and voltage, leading to significant power fluctuations of approximately 174.4 W and 26 W, respectively. Notably, the PSO method shows the lowest power ripple, maintaining a stable output of 0.01 W throughout the duration. Figure 23 highlights a substantial difference in output power between the proposed techniques across varying temperatures. All MPPT methods successfully extracted maximum power at 55 °C, whereas the PWM method extracted the minimum output power, which fell to 20 W. This occurred because, as temperature increases, the optimal voltage decreases, yet the PWM technique maintained a constant output voltage. Figure 23 also showsthe convergence speed of the proposed methods under different scenarios, where FLC takes 0.001 s for settling time, PWM-PI takes 0.07 s, PSO takes 0.1 s and 0.15 s for ANN, and P&O and INC take 0.25 s.

### Case 2: changing in irradiance

**Fig. 24:**
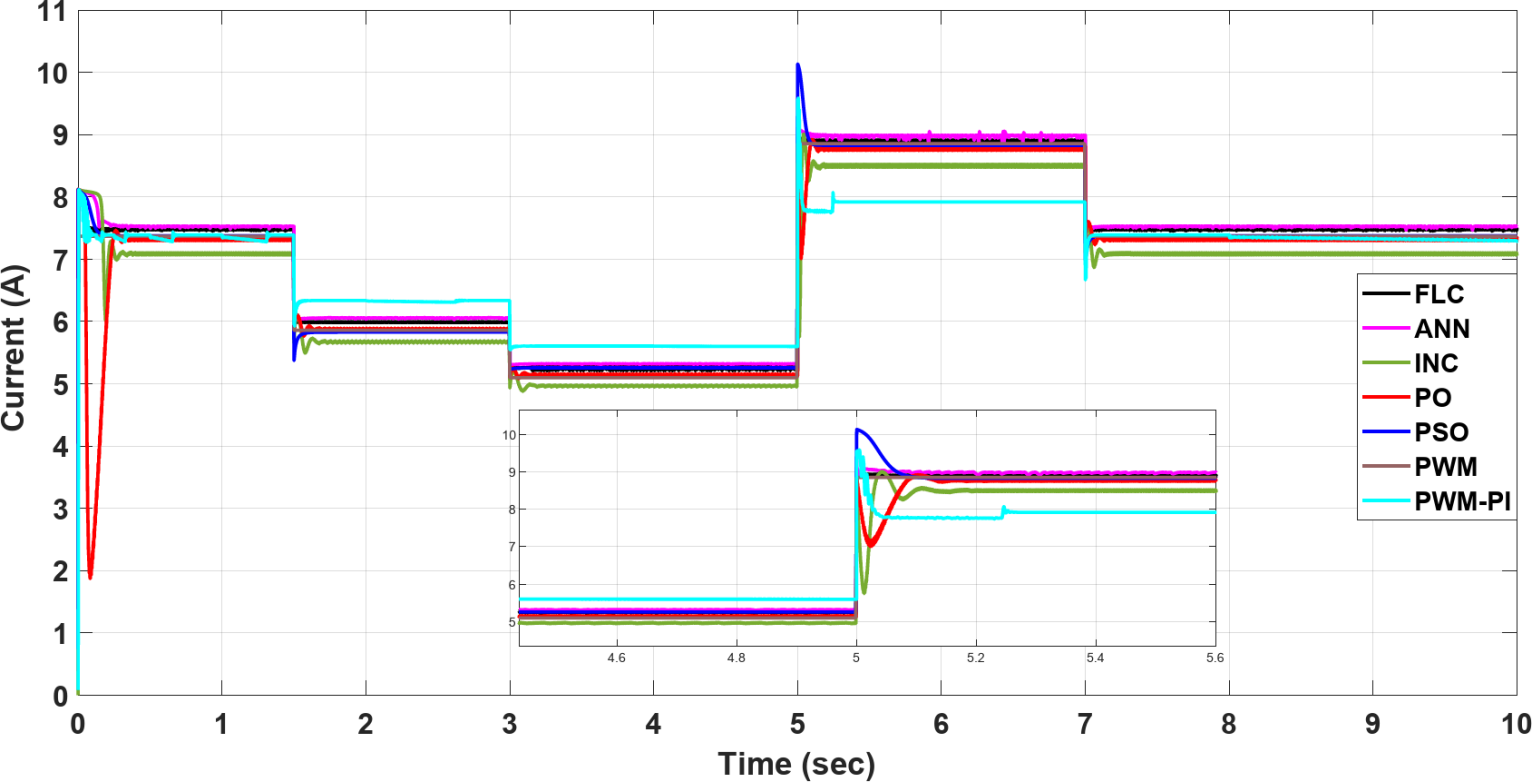
The current profile visirradiance changing.

**Fig. 25:**
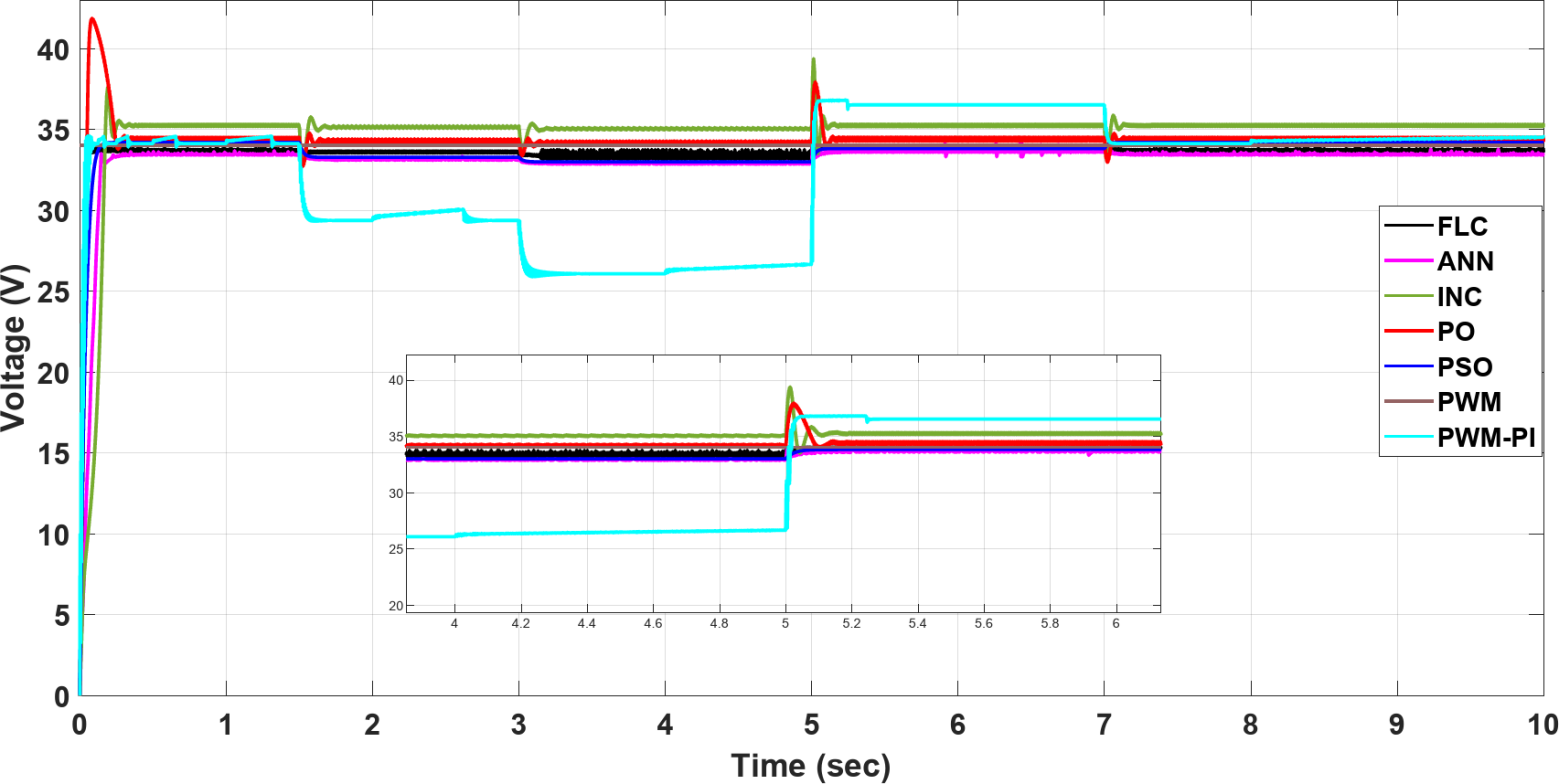
The voltage profile vis irradiance changing.

**Fig. 26:**
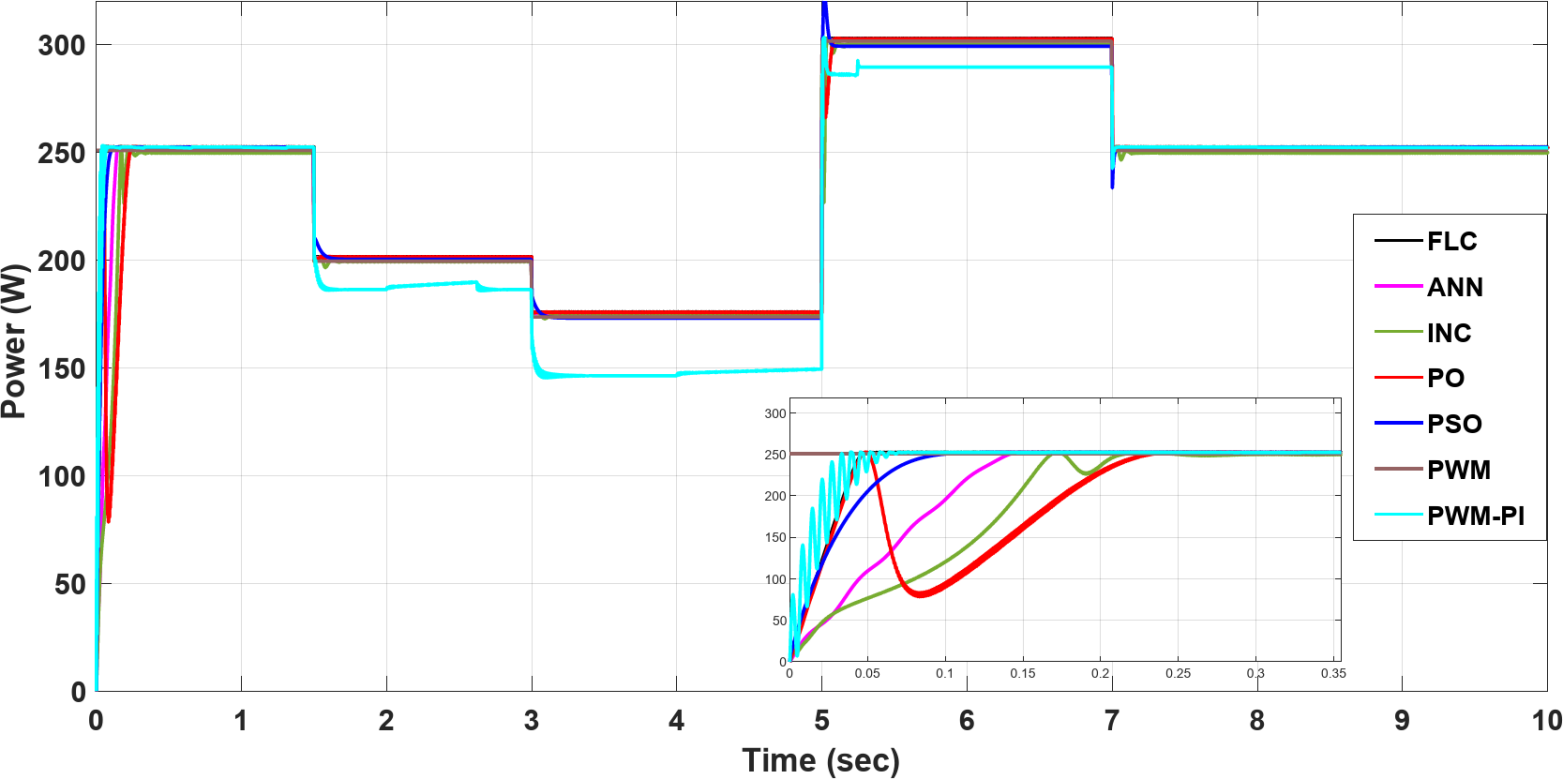
The extracted power response to irradiance changing.

Figures 24, 25 and 26 show that the current varies proportionally with irradiance, while the voltage remains nearly constant throughout, and the power also scales proportionally with irradiance. In further detail, the PWM voltage remains almost constant at $\:{\varvec{V}}_{\varvec{m}}$= 34 V regardless of changes in irradiance, resulting in the current reaching an optimal value of 7.41 A. The P&O and INC show high initial deviations of 5.4 A, while PSO exhibits frequent deviations in current 1.2 A at 5 s, yet maintains a low ripple throughout 0.01 A. Finally, ANN and FLC prove to be the most controlled techniques, maintaining output current close to the optimal value at each change in irradiance, specifically of 5.3 A at 700 W/m2, as shown in Fig. 24. All proposed methods have a good steady-state response, but P&O, INC, and PSO are less efficient transient responses because of high overshooting and undershooting around 5 A and 1.5 A, respectively. On the other hand, the FLC is a more stable method because it can maintain the optimal output current after 0.05 s in contrast to the other proposed methods. Finally, the proportional relationship between current and insolation can be demonstrated by comparing Fig. 20b with Fig. 24, which demonstrates the success of the proposed algorithms with the variation in performance as shown in Fig. 24.

Regarding the voltage profile as mentioned above, the voltage of the PWM technique remains constant at $\:{\varvec{V}}_{\varvec{m}\:}$= 34 V, while the PWM-PI controller’s voltage varies significantly with changing irradiance, reaching its minimum value of 27 V at 700 W/m^2^. P&O and INC exhibit the highest deviations initially, and with each change in irradiance, they show the highest voltage values throughout. The PSO, ANN, and FLC are more stable methods because they can maintain the output current at an optimal stable level of nearly 7.4 A and keep it almost constant, as shown in Fig. 25.

The output power is constantly approaching the optimum values as the PWM voltage remains constant at $\:{\varvec{V}}_{\varvec{m}}$= 34 V throughout the entire process. In contrast, the output power of the PWM-PI controller fluctuates minimally at every irradiance change due to significant voltage variations. Except for the initial high deviations (where P&O and INC exhibit high shooting), all techniques maintain nearly optimal power output without ripple or significant deviations at each irradiance change. The speed convergence of the proposed methods is FLC, PWM-PI, PSO, ANN, INC, and P&O, respectively. The locus of voltage at maximum power varies only slightly, leading to minimal differences between controlled and uncontrolled techniques in terms of output power, as shown in Fig. 26.

### Case 3: simultaneously changing temperature and irradiance

**Fig. 27:**
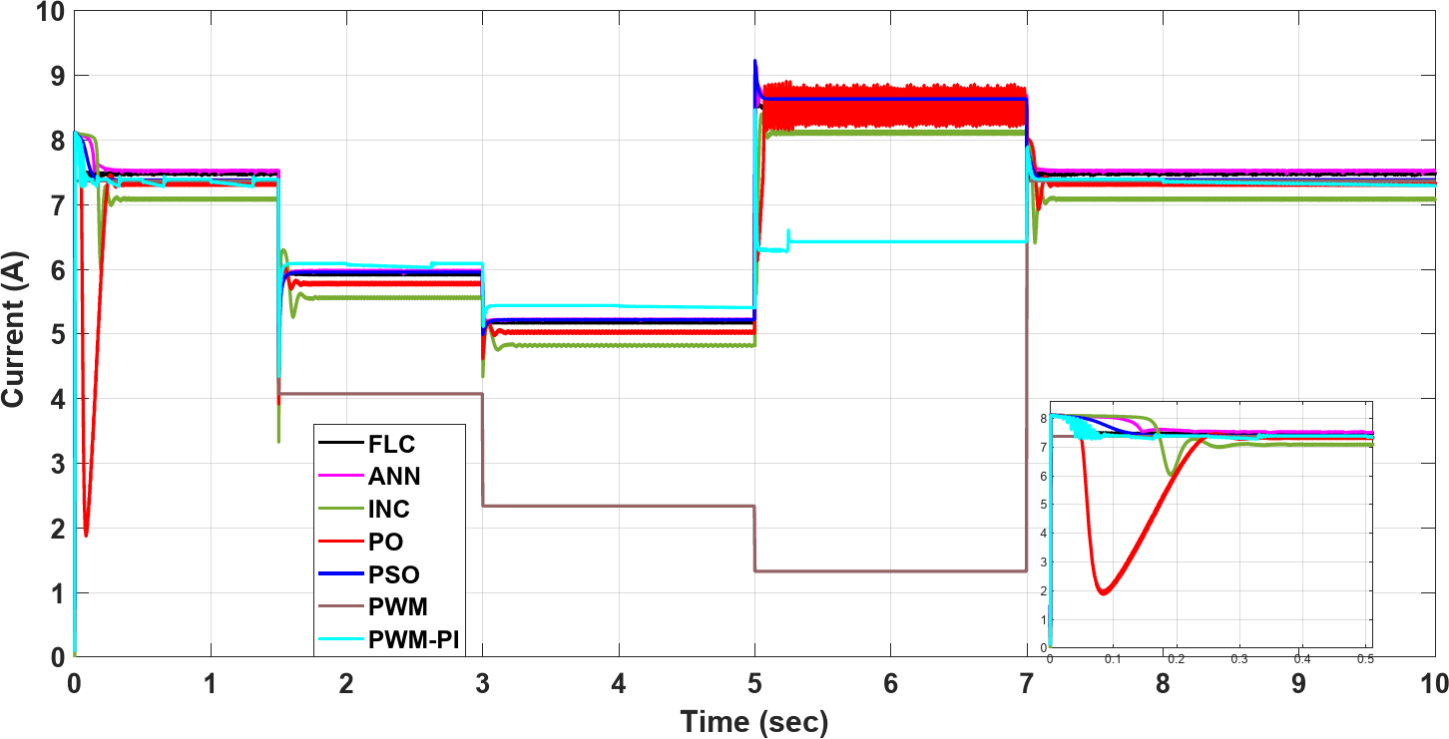
The current profile vis temperature and irradiance changing.

**Fig. 28:**
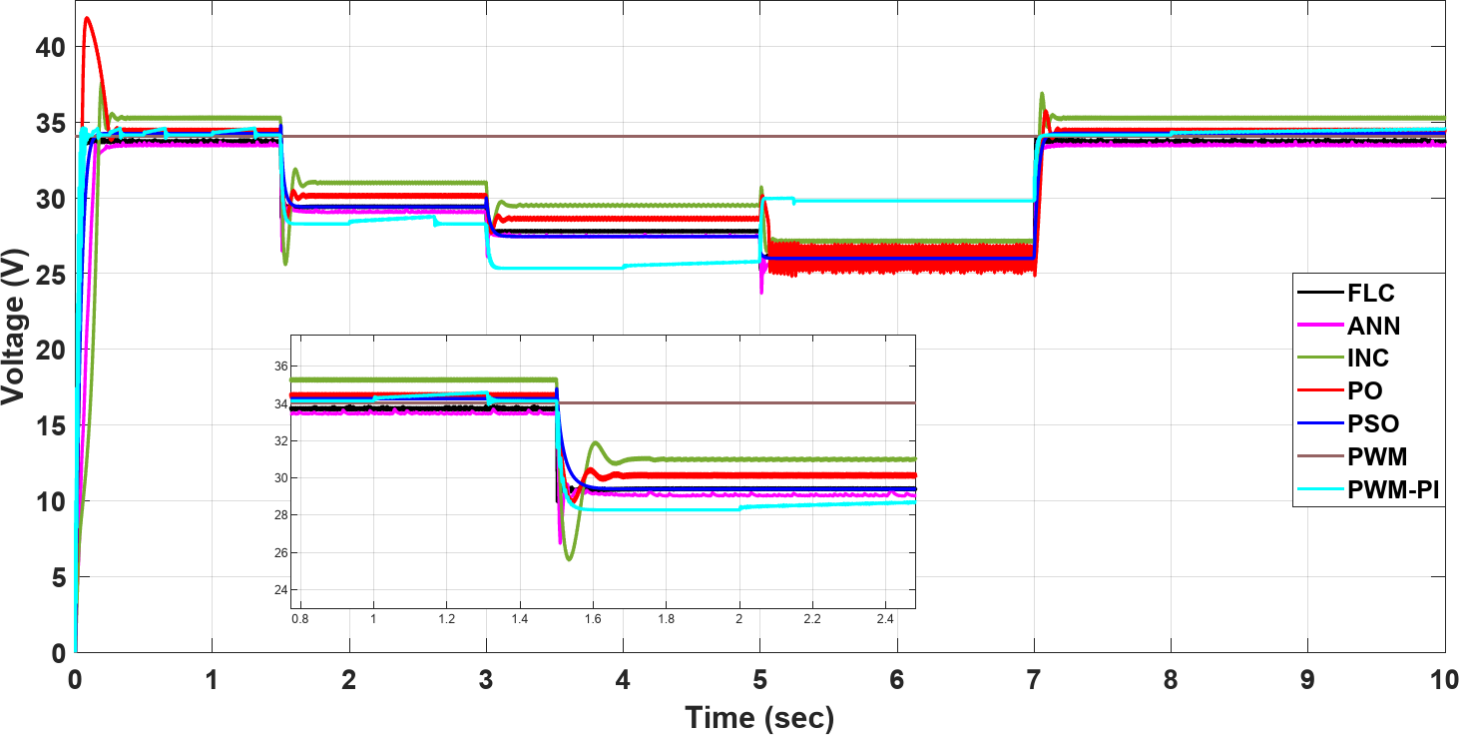
The voltage profile vis temperature and irradiance changing.

**Fig. 29:**
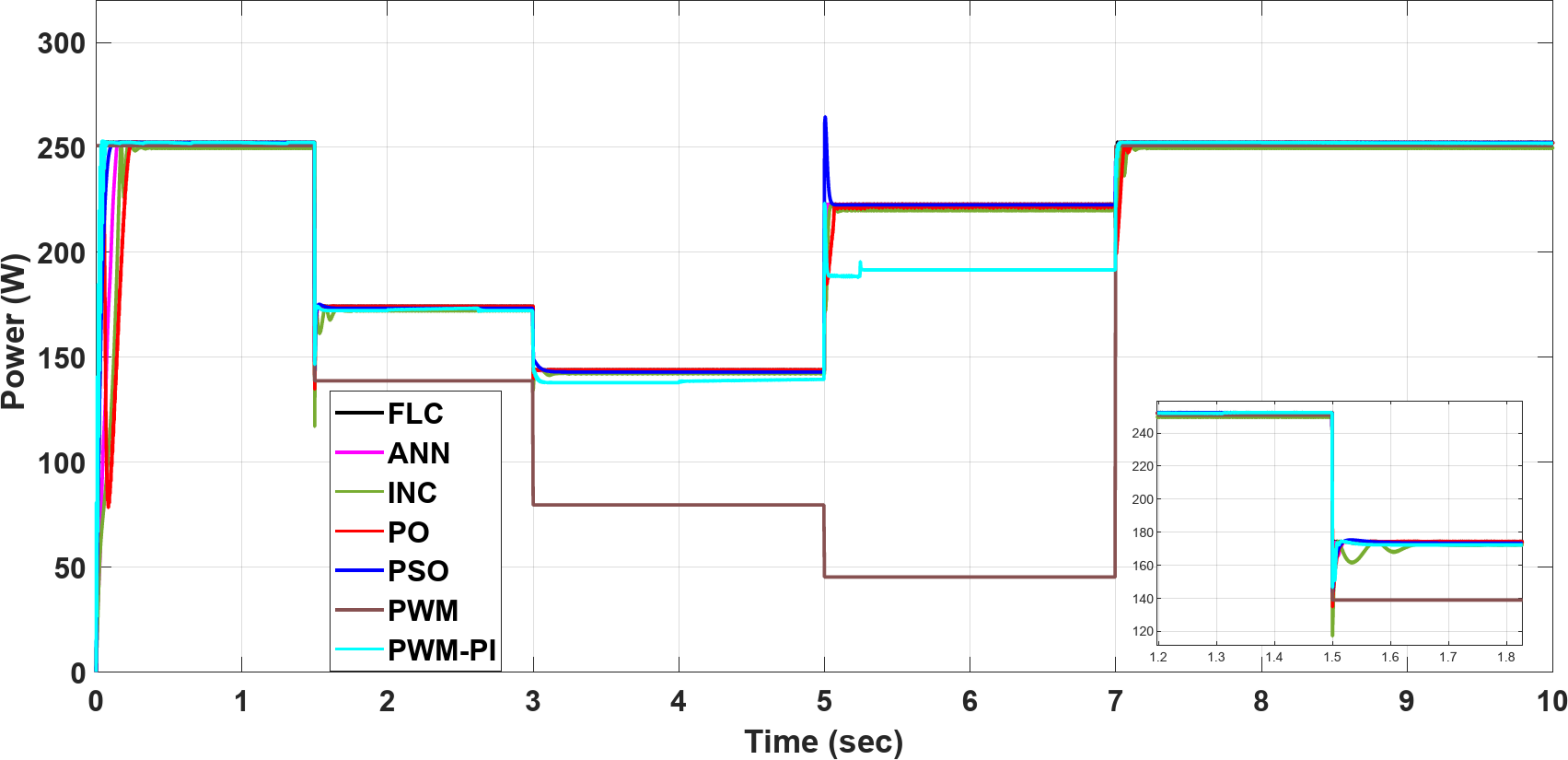
The extracted power response to temperature and irradiance changing.

By combining the simultaneous effects of temperature and irradiance, the resulting current, voltage, and power are presented in Figs. 27, 28 and 29, respectively. According to outcomes, when there are excessive multiple rapid changes in environmental conditions, traditional methods such as P&O and INC are not suitable because of high voltage and current fluctuations, unlike other proposed methods. The results confirmed the superiority of the FLC method over other proposed methods through five standard metrics.

When operating the PWM technique at a constant voltage of $\:{V}_{m}$= 34 V, the system achieves maximum power instantly at 252.41 W. However, without a PI controller, the power output drops significantly to 45.2 W under changing conditions. In contrast, the PWM-PI controller maintains power output near the optimal level of 252.31 W with a minimal ripple of 0.01 W and reduces the drop to 190 W instead of 45.2 W for the same conditions. This method achieves maximum power within a very short duration of 5 ms. The P&O technique exhibits a larger power ripple of 0.5 W, significant undershooting up to 174.4 W, and takes a relatively longer duration of 25 ms to reach maximum power. The INC method, on the other hand, shows a smaller power ripple of 0.3 W, minimal undershooting of 26 W, and achieves maximum power in a shorter duration of 22 ms. PSO techniques demonstrate superior performance with an almost negligible power ripple of 0.001 W, achieving maximum power efficiently. ANN also performs strongly, with a small power ripple of 0.2 W and minimal undershooting of 2.2 W. The FLC method stands out with a very small power ripple of 0.15 W and almost negligible undershooting of 0.5 W, achieving the highest controlled power output of 252.4 W among all methods considered, as shown in Table 6.

**Table 6:** The comparison performance in case 3 for the proposed control methods.

Methods	PWM_PI	P&O	INC	PSO	ANN	FLC
Index						
Max power(W)	252.31	251.6	251.9	252.2	252.2	252.4
Min power(W)	137.3	142	142.3	142.9	143.6	143.9
Settling time (m sec)	7	25	22	12	15	5
Ripple (W)	± 0.01	± 0.5	± 0.3	± 0.001	± 0.2	± 0.15
Shooting (W)	1.6	174.4	26	0.5	2.2	0.5

### Ranking of proposed MPPT methods based on MCDM

Since this paper proposes seven methods and studies their performance under different environmental conditions, it had to be to find the best-performing method. The concept of MCDM was applied through different methods. Firstly, computing the weights for MPPT methods based on different criteria (max power, min power, settling time, shooting, ripple). Then, the VIKOR method is used for ranking the methods and recommending the best method.

#### Computing the weights for MPPT criteria based on CRITIC

All regulated approaches’ performances are compared using the CRITIC methodology. After normalizing the initial data from Table 6, which displays the matrix of decisions, the standard deviation is computed and displayed in Table 7. Next, as indicated in Table 8, the correlation coefficient is determined. Subsequently, each criterion’s weight is determined, as indicated in Table 9.

**Table 7:** CRITIC method-based normalized decision matrix.

	Max power	Min power	Settling time	Ripple	Shooting
PWM_PI	0.8875	0	0.9	0.981964	0.910868
P&O	0	0.712121	0	0	0
INC	0.375	0.757576	0.15	0.400802	0.853364
PSO	0.75	0.848485	0.65	1	1
ANN	0.75	0.954545	0.5	0.601202	0.876366
FLC	1	1	1	0.701403	1

**Table 8:** Values of criteria correlation coefficient.

	Max power	Min power	Settling time	Ripple	Shooting
Max power	1	−0.06308	0.955066	0.870598	0.875036
Min power	−0.06308	1	−0.18653	−0.28486	0.038212
Settling time	0.955066	−0.18653	1	0.834549	0.731479
Ripple	0.870598	−0.28486	0.834549	1	0.846682
Shooting	0.875036	0.038212	0.731479	0.846682	1

**Table 9:** Criteria weights.

	Max power	Min power	Settling time	Ripple	Shooting
$\:{\text{C}}_{\text{j}}$	0.508	1.645	0.665	0.655	0.579
$\:{\text{W}}_{\text{j}}$	0.1252	0.406	0.1642	0.1617	0.1429

#### Computing the weights for MPPT criteria based on AHP

The first step is building the decision matrix based on experts’ opinions as shown in Table 10. Then, using the AHP methodology, the weight for each of the MPPT criteria can be obtained (see Table 11) by evaluating the pairwise comparison matrix (PCM) across the previous indices according to the importance of each as shown in Table 10.

**Table 10:** Decision matrix corresponding to changing of temperature and irradiance simultaneously.

	Max power	Min power	Settling time	Ripple	Shooting
Max power	1	2	3	5	7
Min power	0.5	1	4	5	7
Settling time	0.333333	0.25	1	3	7
Ripple	0.2	0.2	0.333333	1	5
Shooting	0.142857	0.142857	0.142857	0.2	1

**Table 11:** Weight matrix and value of attributes.

	Max power	Min power	Settling time	Ripple	Shooting
W_AHP ($\:{W}_{A})$	0.399702	0.330482	0.155824	0.081597	0.032394
$\:{\lambda\:}_{max}$	5.411321	$\:CR$	0.091441	Consistency check	Passed

#### Evaluation of obtained weights

**Fig. 30:**
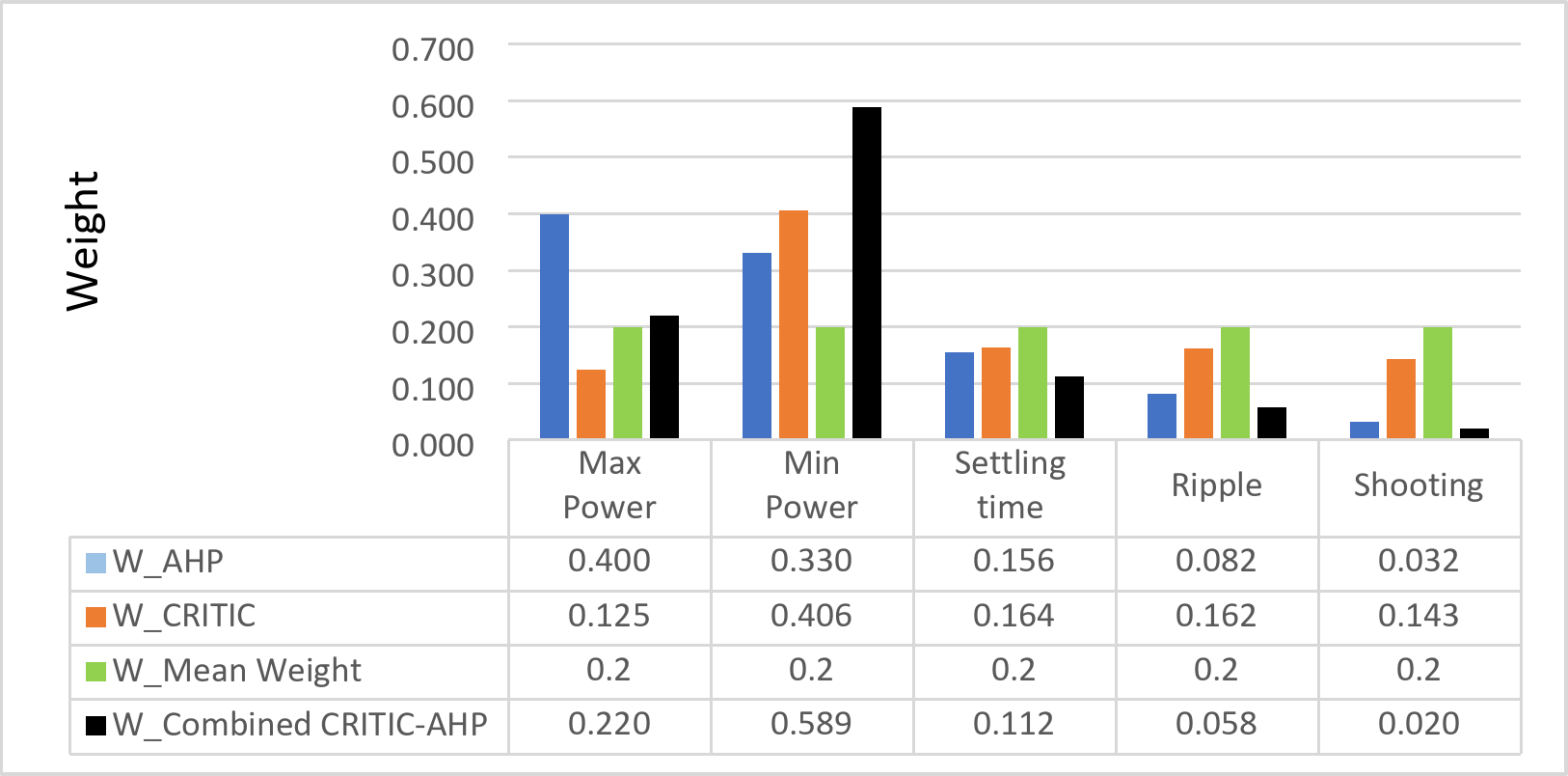
Weight comparison chart.

The MW method and the AHP method, two more MCDM techniques, are contrasted and assessed using the weights that were acquired using the CRITIC approach. To determine the subjective weight of each criterion, an AHP technique is employed, which is a subjective process. The MW approach uses$\:{W}_{j}=\frac{1}{n}$, where $\:n$ is the number of criteria, to determine the objective weight of the criteria. This assumes that every criterion is equally important. When there is insufficient information to make a choice or when the decision maker does not provide information, it is utilized in MCDM^41^. Thecombinedweight $\:{W}_{h}$ can be calculated (see Table 12; Fig. 30).

Figure 30 illustrates how the MW method assigns equal weight to each MPPT criterion. When using the AHP method, variations in the indicators might be reflected in the weight distribution across the criteria. The maximum power weight is demonstrated to be the highest, followed by the minimum power and finally the settling time. Furthermore, ripple and shooting have very low importance.

Table 12 lists the MPPT indices in order according to the weights determined by the AHP subjective weighing approach, the CRITIC and MW objective weighting methods, and a combined AHP-CRITIC method. The combined weight $\:{W}_{h}$is calculated, and it gives higher importance for minimum and maximum power, respectively.

**Table 12:** Comparison of the MPPT criteria ranking.

	Max power	Min power	Settling time	Ripple	Shooting
MW	1	1	1	1	1
CRITIC	1	5	4	3	2
AHP	5	4	3	2	1
CRITIC-AHP	4	5	3	2	1

#### VIKOR ranking method

In the current study, six alternatives are evaluated using five criteria for various power quality phenomena readings using the popular MCDM VIKOR ranking approach. First, as indicated in Table 13, the weighted normalized matrix $\:{wx}_{new}$ is produced to rank alternatives.

**Table 13:** The weighted normalized matrix.

	Max power	Min power	Settling time	Ripple	Shooting
PWM_PI	0.014089	0.405973	0.016422	0.002916	0.012737
P&O	0.125238	0.116871	0.164216	0.161677	0.142897
INC	0.078274	0.098418	0.139584	0.096876	0.020954
PSO	0.031309	0.061511	0.057476	0	0
ANN	0.031309	0.018453	0.082108	0.064476	0.017667
FLC	0	0	0	0.048276	0

Based on CRITIC weights, Mean Weight, AHP and hybrid AHP-CRITIC methods, then $\:{R}_{i}$, $\:{S}_{i}$ and $\:{Q}_{i\:}$are calculated. Following that, three alternate ranking lists are created and sorted according to Table 14 utilizing$\:{S}_{i}$, $\:{R}_{i}$and $\:{Q}_{i}$(for *v* = 0.50) values.

**Table 14:** The combined MCDM-VIKOR method (*v* = 0.5) results.

	CRITIC-VIKOR				Mean weight-VIKOR				AHP-VIKOR				CRITIC & AHP-VIKOR			
	*S*_i_	*R*_i_	*Q*_i_	Rank	*S*_i_	*R*_i_	*Q*_i_	Rank	*S*_i_	*R*_i_	*Q*_i_	Rank	*S*_i_	*R*_i_	*Q*_i_	Rank
PWM_PI	0.452	0.406	0.195	1	0.264	0.200	0.372	3	0.395	0.331	0.342	2	0.6278	0.589	0	1
P&O	0.711	0.164	0.338	2	0.858	0.200	0.000	1	0.765	0.400	0.000	1	0.5796	0.22	0.362198	2
INC	0.434	0.140	0.581	3	0.493	0.170	0.336	2	0.516	0.250	0.368	3	0.4132	0.1428	0.565997	3
PSO	0.150	0.062	0.905	5	0.150	0.070	0.907	5	0.205	0.100	0.778	5	0.1834	0.0892	0.801081	4
ANN	0.214	0.082	0.828	4	0.264	0.100	0.729	4	0.229	0.100	0.761	4	0.1634	0.056	0.846498	5
FLC	0.048	0.048	1.000	6	0.060	0.060	1.000	6	0.024	0.024	1.000	6	0.0173	0.0173	1	6

**Fig. 31:**
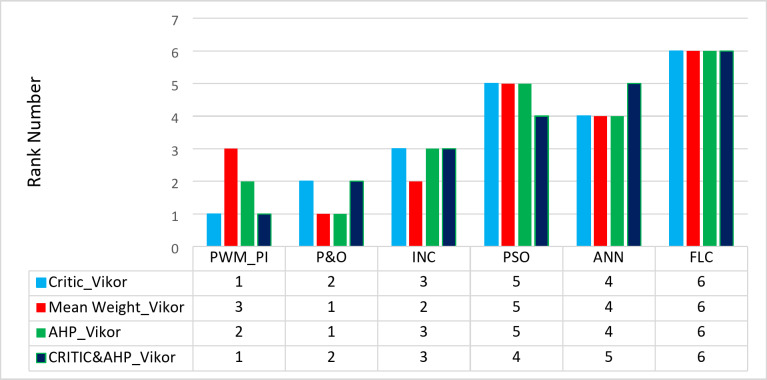
Rank comparison chart.

Based on the ranking values acquired from VIKOR using various weighting methods, Fig. 31 shows aranking of the optimization methodologies under study. All methods agreed that an FLC technique is the best for solving the MPPT problem under different environmental conditions.

All the proposed methods for various parametric variations are compared and summarized in Table 15^42^. It is observed that the FLC demonstrates superior efficiency compared to other methods, with the longest convergence time and the ability to track the MPP more effectively than other algorithms.

**Table 15:** Comparative analysis of the proposed methods.

	Perturb & observe (P&O)	Incremental conductance	Fuzzy logic	Neural networks	PSO
Complexity	Low	Moderate	High	Very High	High
Accuracy & adaptability	Low	Moderate	Excellent	Very High	Very high
Dynamic performance	Low	Moderate	Excellent	Very High	Very high
Oscillations	High	Low	Very low	Low	NON
Computational demand	Low	Moderate	Moderate-high	High	High
Input variables	Voltage & current	Voltage & current	Voltage & current	Voltage & current	Voltage & current
Hardware requirements	Basic: voltage, current sensors, microcontrollers such as Arduino or STM32	Moderate: voltage, current sensors, microcontrollers such as Arduino or STM32	High: DSP/FPGA or Advanced MCU	High: DSP/FPGA	Microcontroller /DSP/FPGA
Cost	Cheap	Moderate	Expensive	Expensive	Moderate
Use cases	Stable environmental conditions	Varying environmental conditions	Rapidly changing environments	Rapidly changing environments	Highly variable conditions
Application	Small-scale PV systems	Medium-scale PV systems.	Large-scale PV systems	Large-scale PV systems	Large-scale PV systems

## Conclusion

Due to the inherent non-linearity of photovoltaic (PV) characteristics, an efficient maximum power point tracking (MPPT) strategy is essential to distinguish the global maximum power point from local optima and maximize energy extraction. This study first presents a comparative analysis of various solar charge regulation techniques in terms of effectiveness and cost. The results indicate that MPPT controllers outperform conventional pulse-width modulation (PWM) controllers by approximately 39% under diverse environmental conditions. Several MPPT techniques, including conventional approaches (Perturb and Observe (P&O), Incremental Conductance (INC)), metaheuristic algorithms (Particle Swarm Optimization (PSO)), and artificial intelligence (AI)-based methods (Artificial Neural Networks (ANN), Fuzzy Logic Control (FLC)), were evaluated to optimize PV energy harvesting.

A comparative assessment of these MPPT algorithms was conducted using a MATLAB-based PV circuit model. The findings demonstrate that traditional techniques such as P&O exhibit power oscillations, overshoot, undershoot, and extended settling time. In contrast, INC achieves lower power fluctuations and reduced transient response time compared to P&O. Among metaheuristic approaches, PSO provides an effective balance between exploration and exploitation, enabling rapid convergence to the maximum power point with minimal power ripple, negligible overshoot, and improved stability. AI-based techniques, particularly FLC, prove highly effective in handling uncertainties and dynamic environmental conditions, leading to enhanced decision-making capabilities in MPPT applications.

To systematically identify the optimal MPPT method, multi-criteria decision-making (MCDM) techniques incorporating weighting methods (Analytic Hierarchy Process (AHP), CRITIC) and a ranking approach (VIKOR) were applied. The simulation results confirm that the FLC-based MPPT technique delivers superior performance, achieving maximum power extraction within the shortest time while improving the overall efficiency of PV systems under varying environmental conditions.

This study presents several key advantages: (1) a comprehensive and objective evaluation of MPPT techniques based on diverse literature sources, (2) enhancement of PV system economic feasibility by maximizing energy yield and minimizing oversizing requirements, (3) identification of the most effective MPPT algorithms under different operational scenarios, (4) standardization of performance comparison metrics using MCDM methodologies, (5) facilitation of future research by consolidating existing techniques, and (6) identification of research gaps, providing insights for further advancements in MPPT technologies. However, certain limitations remain, including (1) the need for a more detailed analysis of MPPT algorithm performance under partial shading conditions, and (2) consideration of real-world implementation challenges such as hardware compatibility, computational complexity, and cost constraints.

Future research directions may focus on hybridizing multiple MPPT strategies to enhance overall performance and achieve global optimization across diverse applications, including PV parameter estimation, load and energy management, PI controller tuning, and integration into conventional and smart grids. Given its critical role in renewable energy systems, MPPT remains a significant and evolving research domain, with potential extensions to other renewable energy sources to further advance sustainable energy solutions.

## Data Availability

The datasets used and/or analysis during the current study available from the corresponding author on reasonable request.

## Abbreviations

ABC: Artificial bee colony
ACO: Ant colony optimizer
AHP: Analytic hierarchy process
AI: Artificial intelligence
ANN: Artificial neural network
ANFIS: Adaptive neuro-fuzzy inference system
COA: Center of area
COG: Center of gravity
CSA: Cuckoo search algorithm
CRITIC: Criteria importance through intercriteria correlation
CV: Constant voltage
EPO: Emperor penguin optimization
FA: Firefly algorithm
FLC: Fuzzy logic controller
FOCV: Fractional open-circuit voltage
FSCC: Fractional short-circuit current
CRITIC: Criteria importance through intercriteria correlation
FSSO: Flying squirrel search optimization
GWO: Gray wolf optimization
GMPP: Global maximum power point
HC: Hill climbing
INC: Incremental conductance
INR: Incremental resistance
JA: Jaya algorithm
MPPT: Maximum power point tracking
MW: Mean weight
MCM: Max criterion method
MOSFET: Metal-oxide-semiconductor field-effect transistor
MCDM: Multi-criteria decision making
MF: Membership function
NM: Negative medium
NS: Negative small
NB: Negative big
PB: Positive big
PI: Proportional and integral
PM: Positive medium
PS: Positive small
PSCs: Partial shading conditions
PSO: Particle swarm optimization
PV: Photovoltaic
PWM: Pulse width modulation
P&O: Perturb and observe
RBFN: Radial basis function network
SSA: Salp swarm algorithms
STC: Standard test condition
ZE: Zero

## List of symbols

$\:{A}^{1}and\:{A}^{2}$: The first- and second-best alternative in the $\:Q$ ranking list
$\:{c}_{1},{c}_{2}$: Cognitive and social parameters
$$\:C$$: Accelerating factor
$$\:CI$$: Capacitor
$$\:{C}_{j}$$: Consistency index
$\:CR$: The information amount in the $\:{j}_{th}$ index
$$\:D$$: Consistency ratio
$$\:{D}_{max},{D}_{min}$$: Duty cycle
$\:{E}_{go}$: Max & min duty cycle
f: The band gap for silicon
$$\:{f}_{j}^{*},\:{f}_{j}^{-}$$: Frequency
$\:{G}_{best}$: The best & worst value
$\:{I}_{0}$: Global best position of particle
$\:{I}_{ph}$: Diode’s saturation current
$\:{I}_{rs}$: Generated PV current
$\:{I}_{sc}$: Reverse saturation current
$\:{I}_{sh}$: Short circuit current
$$\:L$$: Shunt current
$$\:K$$: Inductance
$$\:{K}_{i}$$: Boltzmann’s constant
$\:N$: Temp. coefficient for$\:{I}_{sc}$
$\:{N}_{p}$: The quantity of values of each criterion
$\:{N}_{s}$: No. cell in parallel
$\:{\rho\:}_{jk}$: No. cell in series
$\:P$: Correlation between pairs of criteria
$\:{P}_{best}$: Personal best position of particle
$$\:{P}_{in}$$: Input power
$\:{P}_{PV}$: Extracted power from the PV panel
$$\:q$$: Electron charge
$\:{Q}_{i}$: Alternative ranking indicator
$\:{r}_{ij}$: Normalized matrix of alternative $\:i$ for criterion$\:j$
$\:RI$: The random consistency index
$\:{R}_{i}$: Regret measure values
$$\:{R}_{s}$$: Series resistance
$$\:{R}_{\text{s}\text{h}}$$: Shunt resistance
$\:{S}_{i}$: The utility measure values
$\:T$: Temperature in Kelvin
$$\:{T}_{n}$$: Nominal temp
$\:{v}_{i}^{k}$: Velocity of *i*_*th*_ particle at iteration k
$\:{V}_{d}$: Voltage across the diode
$\:{V}_{o}$: The output voltage
$$\:{V}_{pv}$$: PV voltage
$\:{V}_{oc}$: Open circuit voltage
$$\:w$$: Weight factor
$\:{v}_{i}^{k}$: Position of *i*_*th*_ particle at iteration$\:k$
$\:\varDelta\:D$: Change of duty cycle
$\:\varDelta\:E\left(k\right)$: Change of error
$$\:{\sigma\:}_{j}$$: Standard deviation
$$\:\lambda\:$$: Eigenvector
